# Novel Pyrrole-Based Hydrazide–Hydrazones: Synthesis, In Vitro Evaluation of Antioxidant and Neuroprotective Activity, In Silico ADME, and DFT Studies

**DOI:** 10.3390/antiox15060666

**Published:** 2026-05-25

**Authors:** Valentin Karatchobanov, Emilio Mateev, Iva Valkova, Magdalena Kondeva-Burdina, Maya Georgieva

**Affiliations:** 1 Department of Pharmaceutical Chemistry, Faculty of Pharmacy, Medical University—Sofia, 2 Dunav Str., 1000 Sofia, Bulgaria; v.karachobanov@pharmfac.mu-sofia.bg (V.K.); e.mateev@pharmfac.mu-sofia.bg (E.M.); 2 Department of Chemistry, Faculty of Pharmacy, Medical University—Sofia, 2 Dunav Str., 1000 Sofia, Bulgaria; ivalkova@pharmfac.mu-sofia.bg; 3 Department of Pharmacology, Pharmacotherapy and Toxicology, Faculty of Pharmacy, Medical University—Sofia, 2 Dunav Str., 1000 Sofia, Bulgaria; mkondeva@pharmfac.mu-sofia.bg

**Keywords:** pyrrole, hydrazide–hydrazone, antioxidants, oxidative stress, neuroprotection, ADME, DFT

## Abstract

Pyrrole-based hydrazide–hydrazones constitute a class of organic compounds that has attracted increasing attention in pharmaceutical research due to their potential application in the treatment of neurodegenerative diseases. In the present study, a series of ten novel pyrrole-containing hydrazide–hydrazones was synthesized. The structures of the newly obtained compounds were characterized by IR spectrophotometry, ^1^H-NMR, and ^13^C-NMR spectroscopy, while their purity was confirmed by mass spectrometry and melting point determination. The antioxidant activity of the synthesized compounds was evaluated in vitro using ABTS, DPPH, FRAP, and a commercial Antioxidant Assay Kit (CS0790). Their neurotoxicity and neuroprotective effects were subsequently assessed in rat brain subcellular fractions. Two of the tested compounds, **VV4** and **VV6**, exhibited the most pronounced antioxidant and neuroprotective effects, along with the lowest levels of neurotoxicity. The key pharmacokinetic parameters of the most active compounds were predicted in silico, and their stability and reactivity were subsequently evaluated using DFT analysis.

## 1. Introduction

Oxidative stress represents a condition of disturbed balance between the production and neutralization of reactive oxygen and nitrogen species (RONSs) [1]. RONSs are generated as a result of physiological aerobic metabolic processes and are highly reactive derivatives of oxygen and nitrogen. They are radical (superoxide anion (O_2_^•−^), hydroxyl radical (^•^OH), peroxyl radicals (ROO^•^), alkoxyl radicals (RO^•^), nitric oxide (^•^NO), and nitrogen dioxide (^•^NO_2_)), and non-radical (hydrogen peroxide (H_2_O_2_), singlet oxygen (^1^O_2_), organic hydroperoxides (ROOH), and peroxynitrite anion (ONOO^−^)) [2,3,4,5,6,7,8,9,10]. RONSs are produced by a number of enzymatic and non-enzymatic systems under the control of cytokines and growth factors. There are a number of exogenous factors that increase the production of RONSs and cause oxidative stress—UV rays, ionizing radiation, heavy metals, pesticides, air pollutants, alcohol consumption, smoking, and the intake of certain medications [11,12,13,14,15,16,17,18,19]. The brain is particularly vulnerable and sensitive to oxidative stress because it is the organ with the highest aerobic metabolic activity in the human body. In addition, the brain contains a large amount of lipids, prone to peroxidation, as well as a high concentration of iron ions [20]. The main sources of RONSs in the brain are mitochondria [21]. Other organelles that play an important role in the pathophysiology of oxidative stress in the CNS are the microsomes due to their involvement in the process of lipid peroxidation [22,23]. Some of the most commonly used models for assessing the impact of oxidative stress, as well as the effects of various antioxidants on the brain, are prepared on rat brain synaptosomes [24,25]. Oxidative stress in the central nervous system (CNS), and specifically in the brain, is involved in the pathogenesis of neurodegenerative diseases such as Alzheimer’s disease (AD), Parkinson’s disease (PD), Huntington’s disease (HD), and amyotrophic lateral sclerosis (ALS) [11,20].

Antioxidants are among the most important groups of neuroprotectors. Neuroprotection is expressed in reducing cellular dysfunction and neuronal cell death. Antioxidants bind RONS, thereby restoring the balance between their production and neutralization, preventing oxidative stress, reducing inflammatory processes, and cellular damage in the CNS [26,27,28,29]. Examples of exogenous antioxidants involved in neuroprotective processes include ascorbic acid, tocopherols, polyphenols, and N-acetylcysteine.

Pyrrole has significant importance in modern pharmaceutical science due to its versatile pharmacophoric properties, enabling the development of diverse bioactive compounds. Numerous clinically used drugs containing a pyrrole core exhibit a wide range of activities—antituberculosis, antimalarial, antimycotic, anthelmintic, antitumor, antibiotics, antiviral, nonsteroidal anti-inflammatory, antihyperlipidemic, and nootropic [30,31,32,33,34,35]. Among pyrrole derivatives, hydrazide–hydrazones represent a particularly promising class, as they demonstrate significant antioxidant and neuroprotective potential [36,37,38,39,40,41]. Hydrazones are a group of substances containing the N=CH bond in their structure. They are a good perspective in modern pharmaceutical science. Recent scientific publications [42] report the synthesis of new hydrazone derivatives with promising anti-cancer activity. Additionally, some compounds with a hydrazone group in their structure possess anti-inflammatory, analgesic, anticonvulsant, antidiabetic, antifungal, antibacterial and antiparasitic, immunomodulatory, and antioxidant activity [43,44]. In recent years, several studies [38,39,40,41] have reported the synthesis of novel pyrrole-containing hydrazide–hydrazones, which exhibit pronounced antioxidant and neuroprotective activity in in vitro models. For the most accurate and detailed description of the pharmacological effect of the substances, it is important to also pay attention to their pharmacokinetics, which largely depends on their physicochemical properties [45].

Thus, the present study focuses on the synthesis of a series of novel pyrrole-containing hydrazide–hydrazones, the in vitro evaluation of their antioxidant and neuroprotective potential, the in silico characterization of their molecular properties by DFT, and the prediction of ADME profiles and drug-likeness of the most promising candidates.

## 2. Materials and Methods

Reactants and solvents were obtained from commercial suppliers (Sigma-Aldrich and Acros, Merck KGaA, Darmstadt, Germany), and used without further purification. For the monitoring of the reactions thin layer chromatography (TLC) was applied. The TLC characteristics were measured on Kieselgel 60, F254 (Darmstadt, Germany) at ambient temperature, employing a mobile phase: chloroform:ethanol (10:1). The melting points were determined by Kruss M5000 (Hamburg, Gemrany) and were uncorrected. The absorbance was measured in a multi-plate reader Synergy 2 (BioTek Instruments, Haverhill, MA, USA). The IR spectra range was registered on a Nicolet iS10 FT-IR spectrometer, Smart iTR adapter (Thermo Fisher Scientific, Waltham, MA, USA). The NMR spectra were registered on Bruker Avance Neo (Biospin GmbH, Rheinstetten, Germany), 600 MHz for ^1^H, and 600 MHz for ^13^C. The mass spectrums were measured on a 6410 Agilent LCMS triple quadrupole mass spectrometer (LC/MS) with an electrospray ionization (ESI) interface (Agilent Technologies, Lexington, MA, USA).

### 2.1. Chemistry

The synthetic scheme of the target hydrazide–hydrazones was accomplished according to a procedure reported by E. Mateev et al. [46].

1,4-Dicarbonyl compound: 0.5 mol metallic sodium, cut into flakes, was dissolved in 100 mL abs. ethanol. An amount of 0.5 mol ethyl acetoacetate was included dropwise. After 30 min, 0.5 mol 2,4′-dibromoacetophenone was included. Conventional heating was applied. The reaction was completed in 12 h, detected by TLC. After that, cold water was added, which dissolved the formed NaBr precipitate. The water layer was removed, and the organic layer was washed with HCl and dried over anhydrous Na_2_SO_4_. After that, the dryer was filtered, and the solvent was evaporated, causing the product to crystallize. The crystals were filtered and washed with hexane.

N-pyrrolylcarboxylic acid: 0.1 mol ethyl 2-acetyl-4-(4-bromophenyl)-4-oxobutanoate and 0.12 mol of the amino acid valine were dissolved in 10 mL of glacial acetic acid. Conventional heating was applied. The reaction was completed in 13 h, detected by TLC. After that, the heating was turned off and the reaction mixture was left to cool to room temperature, upon which crystals were formed. The product was filtered, washed with hexane and recrystallized from ethanol.

An esterification with thionylchloride and ethanol was carried out. SOCl_2_ (0.2 mol) was included dropwise to the N-pyrrolylcarboxylic acid (0.09 mol), and diluted in cold abs. ethanol. After 30 min, the mixture was placed on a conventional heating. The reaction time was 2 h, detected by TLC. Subsequently, the solvent was eliminated using a rotary evaporator and the oil was dissolved in dichloromethane, washed with Na_2_CO_3,_ and then dichloromethane was eliminated using a rotary evaporator. The final ethyl ester of the N-pyrrolylcarboxylic acid was incorporated in the next synthetic phase without isolation.

The hydrazinolysis was carried out by reacting 0.07 mol of the ethyl ester of the N-pyrrolylcarboxylic acid and 0.35 mol hydrazine hydrate in 25 mL of abs. ethanol. Conventional heating was applied. The reaction was completed in 72 h, detected by TLC. After that, crystals were formed. The pyrrole-based hydrazide was filtered, washed with hexane and cold abs. ethanol, and then recrystallized from ethanol.

The target hydrazide–hydrazones (**VV1**–**VV10**) were synthesized using a reaction of the hydrazizde of the N-pyrrolylcarboxylic acid with different aldehydes (**1**–**10**) by conventional heating. Glacial acetic acid was applied as a catalyst and a solvent. The reaction times were between 45 min and 2 h. The completed reactions (detected by TLC) were poured into cold water and the crystals were filtered. The hydrazide–hydrazones were washed with hexane and recrystallized from ethanol/water.

*Ethyl-5-(4-bromophenyl)-1-(1-(2-benzylidenehydrazineyl)-3-methyl-1-oxobutane-2-yl)-2-methyl-1H-pyrrole-3-carboxylate* (**VV1**): Yield 92%; melting point 144.6–145.2 °C; Rf 0.53 (10:0.4); purity > 95% (HPLC); IR ν_max_: 2966 (NH), 1624 (C=O), 1569 (C=N), 775 (p-disubstituted C_6_H_4_), 751 (monosubstituted C_6_H_5_), 552 (Br); ^1^H NMR (DMSO, 600 MHz) δ ppm: 11.20 (1H, s, NH); 8.32 (1H, s, N=CH); 7.90 (2H, d, J = 1.82 Hz, H-2″, H-6″ (benzene)); 7.66 (2H, d, *J* = 8.47 Hz, H-2′, H-6′ (bromobenzene)); 7.62 (1H, s, H-4″ (benzene)); 7.54 (2H, d, *J* = 1.56 Hz, H-3″, H-5″ (benzene)); 7.34 (2H, d, J = 8.46 Hz, H-3″, H-5″ (bromobenzene)); 6.41 (1H, s, H-3 (pyrrole)); 4.34 (1H, d, *J* = 10.50 Hz, CH-CONH); 4.17 (2H, q, J = 7.14 Hz, CH_2_-CH_3_); 2.51 (3H, m, J = 1.75 Hz, 2-CH_3_ (pyrrole)); 1.92 (1H, s, CH-(CH_3_)_2_); 1.26 (3H, t, *J* = 7.14 Hz, CH_2_-CH_3_); 1.07 (3H, d, J = 2.22 Hz, CH_3_ (isopropyl)); 0.94 (3H, d, J = 6.24 Hz, CH_3_ (isopropyl)); ^13^C-NMR (600 MHz) δ ppm: 11.5, 15.7, 18.4, 22.1, 39.8, 59.4, 79.7, 112.5, 116.8, 117.7, 120.5, 121.8, 128.7, 129.5, 132.0, 134.2, 146.6, 148.8, 151.0, 162.1, 164.6; *m*/*z* (FTMS + pESI) 512.05 [M ^+^ 2H^+^].

*Ethyl-5-(4-bromophenyl)-1-(1-(2-(furan-2-ylmethylidene)-hydrazineyl)-3-methyl-1-oxobutane-2-yl)-2-methyl-1H-pyrrole-3-carboxylate* (**VV2**): Yield 96%; melting point 148.8–149.3 °C; Rf 0.57 (10:0.4); purity > 95% (HPLC); IR ν_max_: 2966 (NH), 1633 (C=O), 1568 (C=N), 1010 (furan ring), 774 (p-disubstituted C_6_H_4_), 549 (Br); ^1^H NMR (DMSO, 600 MHz) δ ppm: 11.15 (1H, s, NH); 8.32 (1H, s, N=CH); 7.81 (1H, s, H-5″ (furan)); 7.65 (2H, d, *J* = 8.52 Hz, H-2′, H-6′ (bromobenzene)); 7.32 (2H, d, *J* = 8.49 Hz, H-3′, H-5′ (bromobenzene)); 7.12 (1H, m, *J* = 3.44 Hz, H-3″ (furan)); 6.72 (1H, m, J = 5.26 Hz, H-4″ (furan)); 6.41 (1H, s, H-3 (pyrrole)); 4.31 (1H, d, *J* = 10.71 Hz, CH-CONH); 4.17 (2H, q, *J* = 7.11 Hz, CH_2_-CH_3_); 2.52 (3H, m, *J* = 1.88 Hz, 2-CH_3_ (pyrrole)); 1.92 (1H, s, CH-(CH3)2); 1.26 (3H, t, J = 7.21 Hz, CH2-CH3); 1.06 (3H, d, *J* = 6.96 Hz, CH_3_ (isopropyl)); 0.94 (3H, d, *J* = 6.32 Hz, CH_3_ (isopropyl)); ^13^C-NMR (600 MHz) δ ppm: 11.9, 14.9, 18.7, 21.5, 40.1, 59.4, 79.8, 112.5, 116.8, 117.7, 120.2, 121.8, 131.7, 132.3, 139.8, 142.0, 147.2, 149.4, 151.0, 164.6, 171.5, 172.4; *m*/*z* (FTMS + pESI) 502.76 [M ^+^ 2H^+^].

*Ethyl-5-(4-bromophenyl)-1-(1-(2-(2,4-dihydroxybenzylidene)-hydrazineyl)-3-methyl-1-oxobutane-2-yl)-2-methyl-1H-pyrrole-3-carboxylate* (**VV3**): Yield 95%; melting point 158.3–158.6 °C; Rf 0.60 (10:0.4); purity > 95% (HPLC); IR ν_max_: 3322 (OH), 2967 (NH), 1615 (C=O), 1557 (C=N), 813 (trisubstituted C_6_H_3_), 774 (p-disubstituted C_6_H_4_), 552 (Br); ^1^H NMR (DMSO, 600 MHz) δ ppm: 11.40 (1H, s, 2″-OH (dihydroxybenzene)); 11.17 (1H, s, NH); 9.82 (1H, s, 4″-OH (dihydroxybenzene)); 8.32 (1H, s, N=CH); 7.66 (2H, d, *J* = 8.44 Hz, H-2′, H-6′ (bromobenzene)); 7.44 (1H, s, H-6″ (dihydroxybenzene)); 7.42 (1H, s, H-3″ (dihydroxybenzene)); 7.31 (2H, d, *J* = 8.48 Hz, H-3′, H-5′ (bromobenzene)); 6.41 (1H, s, H-3 (pyrrole)); 6.34 (1H, d, *J* = 2.39 Hz, H-5″ (dihydroxybenzene)); 4.32 (1H, d, *J* = 10.73 Hz, CH-CONH); 4.17 (2H, q, *J* = 7.10 Hz, CH_2_-CH_3_); 2.51 (3H, m, *J* = 1.41 Hz, 2-CH_3_ (pyrrole)); 1.92 (1H, s, CH-(CH_3_)_2_); 1.26 (3H, t, *J* = 7.06 Hz, CH_2_-CH_3_); 1.06 (3H, d, *J* = 6.97 Hz, CH_3_ (isopropyl)); 0.94 (3H, d, *J* = 6.35 Hz, CH_3_ (isopropyl)); ^13^C-NMR (600 MHz) δ ppm: 11.9, 14.9, 19.0, 21.5, 40.1, 59.8, 79.7, 112.1, 116.8, 117.8, 120.2, 121.8, 128.9, 132.0, 134.2, 146.6, 151.6, 164.9, 171.6, 172.5; *m*/*z* (FTMS + pESI) 544.96 [M ^+^ 2H^+^].

*Ethyl-5-(4-bromophenyl)-1-(1-(2-(2,4,6-trihydroxybenzylidene)-hydrazineyl)-3-methyl-1-oxobutane-2-yl)-2-methyl-1H-pyrrole-3-carboxylate* (**VV4**): Yield 97%; melting point 160.8–161.2 °C; Rf 0.57 (10:0.4); purity > 95% (HPLC); IR ν_max_: 3320 (OH), 2969 (NH), 1614 (C=O), 1568 (C=N), 814 (tetrasubstituted C_6_H_2_), 774 (p-disubstituted C_6_H_4_), 560 (Br); ^1^H NMR (DMSO, 600 MHz) δ ppm: 11.18 (1H, s, NH); 9.68 (1H, s, 2″-OH (trihydroxybenzene)); 9.62 (1H, s, 6″-OH (trihydroxybenzene)); 9.49 (1H, s, 4″-OH (trihydroxybenzene)); 8.32 (1H, s, N=CH); 7.68 (2H, d, *J* = 8.45 Hz, H-2′, H-6′ (bromobenzene)); 7.33 (2H, d, *J* = 8.45 Hz, H-3′, H-5′ (bromobenzene)); 7.08 (2H, s, H-3″, H-5″ (trihydroxybenzene)); 6.41 (1H, s, H-3 (pyrrole)); 4.32 (1H, d, *J* = 10.70 Hz, CH-CONH); 4.17 (2H, q, *J* = 7.17 Hz, CH_2_-CH_3_); 2.51 (3H, m, *J* = 1.82 Hz, 2-CH_3_ (pyrrole)); 1.92 (1H, s, CH-(CH_3_)_2_); 1.26 (3H, t, *J* = 7.14 Hz, CH_2_-CH_3_); 1.07 (3H, d, *J* = 6.99 Hz, CH_3_ (isopropyl)); 0.94 (3H, d, *J* = 6.32 Hz, CH_3_ (isopropyl)); ^13^C-NMR (600 MHz) δ ppm: 12.5, 14.6, 19.6, 20.5, 40.1, 59.4, 79.8, 112.8, 116.2, 118.7, 122.1, 131.2, 132.1, 134.2, 146.3, 152.2, 164.6, 171.5, 176.2; *m*/*z* (FTMS + pESI) 560.57 [M + 2H+].

*Ethyl-5-(4-bromophenyl)-1-(1-(2-(3,5-dimethoxy-4-hydroxybenzylidene)-hydrazineyl)-3-methyl-1-oxobutane-2-yl)-2-methyl-1H-pyrrole-3-carboxylate* (**VV5**): Yield 96%; melting point 149.0–149.3 °C; Rf 0.64 (10:0.4); purity > 95% (HPLC); IR ν_max_: 3543 (OCH_3_), 2971 (NH), 1670 (C=O), 1569 (C=N), 817 (tetrasubstituted C_6_H_2_), 775 (p-disubstituted C_6_H_4_), 560 (Br); ^1^H NMR (DMSO, 600 MHz) δ ppm: 11.07 (1H, s, NH); 9.07 (1H, s, 4″-OH (3,5-dimethoxy-4-hydroxybenzene)); 8.32 (1H, s, N=CH); 7.66 (2H, d, *J* = 8.30 Hz, H-2′, H-6′ (bromobenzene)); 7.33 (2H, d, *J* = 8.45 Hz, H-3′, H-5′ (bromobenzene)); 6.94 (2H, d, *J* = 9.86 Hz, H-2″, H-6″ (3,5-dimethoxy-4-hydroxybenzene)); 6.41 (1H, s, H-3 (pyrrole)); 4.32 (1H, d, J = 10.65 Hz, CH-CONH); 4.17 (2H, q, *J* = 7.10 Hz, CH_2_-CH_3_); 3.83 (3H, s, 3″-OCH_3_ (3,5-dimethoxy-4-hydroxybenzene); 3.83 (3H, s, 5″-OCH_3_ (3,5-dimethoxy-4-hydroxybenzene); 2.51 (3H, m, *J* = 1.38 Hz, 2-CH_3_ (pyrrole)); 1.92 (1H, s, CH-(CH_3_)_2_); 1.26 (3H, t, *J* = 7.06 Hz, CH_2_-CH_3_); 1.06 (3H, d, *J* = 7.06 Hz, CH_3_ (isopropyl)); 0.94 (3H, d, *J* = 6.32 Hz, CH_3_ (isopropyl)); ^13^C-NMR (600 MHz) δ ppm: 12.5, 14.9, 19.0, 21.4, 40.1, 56.2, 59.4, 79.6, 106.2, 110.0, 116.2, 122.1, 125.2, 132.3, 139.5, 148.5, 161.2, 164.9, 171.5, 172.4; *m*/*z* (FTMS + pESI) 588.18 [M ^+^ 2H^+^].

*Ethyl-5-(4-bromophenyl)-1-(1-(2-(3-methoxy-4-hydroxybenzylidene)-hydrazineyl)-3-methyl-1-oxobutane-2-yl)-2-methyl-1H-pyrrole-3-carboxylate* (**VV6**): Yield 97%; melting point 156.8–157.3 °C; Rf 0.58 (10:0.4); purity > 95% (HPLC); IR ν_max_: 2968 (NH), 1667 (C=O), 1569 (C=N), 817 (trisubstituted C_6_H_3_), 774 (p-disubstituted C_6_H_4_), 555 (Br); ^1^H NMR (DMSO, 600 MHz) δ ppm: 11.02 (1H, s, NH); 9.62 (1H, s, 4″-OH (3-methoxy-4-hydroxybenzene)); 8.32 (1H, s, N=CH); 7.68 (2H, d, *J* = 8.36 Hz, H-2′, H-6′ (bromobenzene)); 7.33 (2H, d, *J* = 8.50 Hz, H-3′, H-5′ (bromobenzene)); 6.89 (1H, s, H-2″ (3-methoxy-4-hydroxybenzene)); 6.87 (1H, s, H-6″ (3-methoxy-4-hydroxybenzene)); 6.59 (1H, s, H-5″ (3-methoxy-4-hydroxybenzene)); 6.41 (1H, s, H-3 (pyrrole)); 4.32 ((1H, d, *J* = 10.71 Hz, CH-CONH)); 4.17 (2H, q, *J* = 7.10 Hz, CH_2_-CH_3_); 3.84 (3H, s, 3″-OCH_3_); 2.51 (3H, m, *J* = 1.34 Hz, 2-CH_3_ (pyrrole)); 1.92 (1H, s, CH-(CH_3_)_2_); 1.26 (3H, t, *J* = 7.17 Hz, CH_2_-CH_3_); 1.06 (3H, d, *J* = 7.06 Hz, CH_3_ (isopropyl)); 0.94 (3H, d, *J* = 6.32 Hz, CH_3_ (isopropyl)); ^13^C-NMR (600 MHz) δ ppm: 12.5, 14.9, 19.0, 21.5, 40.1, 56.3, 59.4, 79.6, 106.2, 110.0, 116.2, 122.1, 126.4, 132.3, 139.6, 148.6, 161.8, 164.9, 171.2, 172.1; *m*/*z* (FTMS + pESI) 558.32 [M ^+^ 2H^+^].

*Ethyl-5-(4-bromophenyl)-1-(1-(2-(2-fluorobenzylidene)-hydrazineyl)-3-methyl-1-oxobutane-2-yl)-2-methyl-1H-pyrrole-3-carboxylate* (**VV7**): Yield 96%; melting point 150.4–151.0 °C; Rf 0.53 (10:0.4); purity > 95% (HPLC); IR ν_max_: 2964 (NH), 1625 (C=O), 1572 (C=N), 958 (F), 774 (p-disubstituted C_6_H_4_), 751 (o-disubstituted C_6_H_4_), 541 (Br); ^1^H NMR (DMSO, 600 MHz) δ ppm: 11.35 (1H, s, NH); 8.32 (1H, s, N=CH); 7.70 (1H, s, H-6″ (2-fluorobenzene)); 7.66 (2H, d, *J* = 8.44 Hz, H-2′, H-6′ (bromobenzene)); 7.43 (1H, d, *J* = 8.66 Hz, H-4″ (2-fluorobenzene)); 7.33 (2H, d, *J* = 8.51 Hz, H-3′, H-5′ (bromobenzene)); 7.30 (1H, d, *J* = 2.50 Hz, H-5” (2-fluorobenzene)); 7.28 (1H, s, H-3″ (2-fluorobenzene)); 6.41 (1H, s, H-3 (pyrrole)); 4.32 (1H, d, *J* = 10.71 Hz, CH-CONH); 4.17 (2H, q, *J* = 7.13 Hz, CH_2_-CH_3_); 2.51 (3H, m, *J* = 1.42 Hz, 2-CH_3_ (pyrrole)); 1.92 (1H, s, CH-(CH_3_)_2_); 1.26 (3H, t, *J* = 7.07 Hz, CH_2_-CH_3_); 1.06 (3H, d, *J* = 7.04 Hz, CH_3_ (isopropyl)); 0.94 (3H, d, *J* = 6.26 Hz, CH_3_ (isopropyl)); ^13^C-NMR (600 MHz) δ ppm: 11.9, 14.0, 19.0, 29.1, 40.1, 59.4, 79.7, 106.9, 110.6, 116.9, 121.8, 125.5, 128.6, 132.3, 134.2, 146.9, 155.6, 160.9, 163.1, 171.2; *m*/*z* (FTMS + pESI) 530.12 [M ^+^ 2H^+^].

*Ethyl-5-(4-bromophenyl)-1-(1-(2-(2-hydroxybenzylidene)-hydrazineyl)-3-methyl-1-oxobutane-2-yl)-2-methyl-1H-pyrrole-3-carboxylate* (**VV8**): Yield 95%; melting point 151.2–151.8 °C; Rf 0.55 (10:0.4); purity > 95% (HPLC); IR ν_max_: 2971 (NH), 1663 (C=O), 1571 (C=N), 782 (p-disubstituted C_6_H_4_), 747 (o-disubstituted C_6_H_4_), 564 (Br); ^1^H NMR (DMSO, 600 MHz) δ ppm: 11.13 (1H, s, 2″-OH (2-hydroxybenzene)); 11.06 (1H, s, NH); 8.32 (1H, s, N=CH); 7.70 (1H, d, *J* = 6.36 Hz, H-6″ 2-hydroxybenzene)); 7.66 (2H, d, *J* = 8.29 Hz, H-2′, H-6′ (bromobenzene)); 7.53 (1H, m, *J* = 7.16 Hz, H-5″ (2-hydroxybenzene)); 7.42 (1H, m, *J* = 7.39 Hz, H-4″ (2-hydroxybenzene)); 7.33 (2H, d, *J* = 8.48 Hz, H-3′, H-5′ (bromobenzene)); 6.98 (1H, d, *J* = 7.55 Hz, H-3″ (2-hydroxybenzene)); 6.41 (1H, s, H-3 (pyrrole)); 4.35 (1H, d, *J* = 10.63 Hz, CH-CONH); 4.17 (2H, q, *J* = 7.10 Hz, CH_2_-CH_3_); 2.51 (3H, m, *J* = 1.85 Hz, 2-CH_3_ (pyrrole)); 1.92 (1H, s, CH-(CH_3_)_2_); 1.26 (3H, t, *J* = 7.06 Hz, CH_2_-CH_3_); 1.06 (3H, d, *J* = 7.02 Hz, CH_3_ (isopropyl)); 0.94 (3H, d, *J* = 6.28 Hz, CH_3_ (isopropyl)); ^13^C-NMR (600 MHz) δ ppm: 11.9, 14.2, 19.0, 29.2, 40.1, 59.7, 79.5, 105.6, 109.7, 115.3, 118.7, 127.4, 131.1, 133.7, 146.3, 152.2, 159.0, 163.4, 173.0; *m*/*z* (FTMS + pESI) 528.73 [M ^+^ 2H^+^].

*Ethyl-5-(4-bromophenyl)-1-(1-(2-(anthracene-9-ylmethylidene)-hydrazineyl)-3-methyl-1-oxobutane-2-yl)-2-methyl-1H-pyrrole-3-carboxylate* (**VV9**): Yield 92%; melting point 152.0–152.4 °C; Rf 0.63 (10:0.4); purity > 95% (HPLC); IR ν_max_: 2971 (NH), 1665 (C=O), 1568 (C=N), 775 (p-disubstituted C_6_H_4_), 727 (anthracene), 559 (Br); ^1^H NMR (DMSO, 600 MHz) δ ppm: 11.45 (1H, s, NH); 8.71 (1H, s, H-10″ (anthracene)); 8.32 (1H, s, N=CH); 8.15 (2H, d, *J* = 8.38 Hz, H-4″, H-5″ (anthracene)); 7.71 (2H, d, *J* = 7.50 Hz, H-1″, H-8″ (anthracene)); 7.66 (2H, d, *J* = 8.34 Hz, H-2′, H-6′ (bromobenzene)); 7.58 (4H, m, *J* = 8.15 Hz, H-2”, H-3″, H-6″, H-7″ (anthracene)); 7.33 (2H, d, *J* = 8.55 Hz, H-3′, H-5′ (bromobenzene)); 6.41 (1H, s, H-3 (pyrrole)); 4.32 (1H, d, *J* = 10.68 Hz, CH-CONH); 4.17 (2H, q, *J* = 7.10 Hz, CH_2_-CH_3_); 2.51 (3H, m, *J* = 1.82 Hz, 2-CH_3_ (pyrrole)); 1.92 (1H, s, CH-(CH_3_)_2_); 1.26 (3H, t, *J* = 7.12 Hz, CH_2_-CH_3_); 1.06 (3H, d, *J* = 6.99 Hz, CH_3_ (isopropyl)); 0.94 (3H, d, *J* = 6.22 Hz, CH_3_ (isopropyl)); ^13^C-NMR (600 MHz) δ ppm: 12.1, 14.9, 19.0, 29.1, 40.1, 59.4, 79.8, 103.1, 107.2, 110.9, 113.4, 122.0, 125.2, 126.1, 127.7, 128.3, 129.8, 131.4, 132.2, 142.0, 150.0, 160.9, 164.6, 171.4; *m*/*z* (FTMS + pESI) 612.54 [M ^+^ 2H^+^].

*Ethyl-5-(4-bromophenyl)-1-(1-(2-((5-nitrofuran-2-yl)methylidene)-hydrazineyl)-3-methyl-1-oxobutane-2-yl)-2-methyl-1H-pyrrole-3-carboxylate* (**VV10**): Yield 97%; melting point 165.1–165.7 °C; Rf 0.50 (10:0.4); purity > 95% (HPLC); IR ν_max_: 2968 (NH), 1698 (C=O), 1567 (C=N), 1348 (NO_2_), 1011 (furan ring), 775 (p-disubstituted C_6_H_4_), 562 (Br); ^1^H NMR (DMSO, 600 MHz) δ ppm: 11.62 (1H, s, NH); 8.32 (1H, s, N=CH); 7.83 (1H, d, *J* = 4.06 Hz, H-4″ (5-nitrofuran)); 7.66 (2H, d, *J* = 8.45 Hz, H-2′, H-6′ (bromobenzene)); 7.33 (2H, d, *J* = 8.33 Hz, H-3′, H-5′ (bromobenzene)); 7.20 (1H, d, *J* = 3.83 Hz, H-3″ (5-nitrofuran)); 6.41 (1H, s, H-3 (pyrrole)); 4.32 (1H, d, *J* = 10.78 Hz, CH-CONH); 4.17 (2H, q, *J* = 7.04 Hz, CH_2_-CH_3_); 2.51 (3H, m, *J* = 2.82 Hz, 2-CH_3_ (pyrrole)); 1.92 (1H, s, CH-(CH_3_)_2_); 1.26 (3H, t, *J* = 7.00 Hz, CH_2_-CH_3_); 1.06 (3H, d, *J* = 6.96 Hz, CH_3_ (isopropyl)); 0.94 (3H, d, J = 6.39 Hz, CH_3_ (isopropyl)); ^13^C-NMR (600 MHz) δ ppm: 12.5, 14.9, 19.0, 28.9, 40.1, 59.4, 79.6, 110.3, 114.8, 116.6, 120.2, 121.9, 130.5, 132.2, 139.5, 140.7, 145.1, 149.1, 150.7, 164.7, 171.5; *m*/*z* (FTMS + pESI) 547.24 [M ^+^ 2H^+^].

### 2.2. Antioxidant Assays

#### 2.2.1. ABTS Assay

The studies were conducted according to a modified method of Arnao et al. [47]. ABTS·^+^ was generated by mixing equal amounts of a 2.4 mmol/L solution of potassium persulfate and a 7 mmol/L solution of ABTS. The resulting methanolic solution was left to react in the dark for 14 h. The working solution was made of 2 mL of the stock solution diluted in 50 mL of methanol. In total, to the ABTS working solution, a methanolic solution of the tested substance at different concentrations was added. The samples were well mixed and incubated for 7 min in the dark. Absorption was measured at a wavelength of 734 nm against a control sample that did not contain a solution of the tested substances. The percentage of ABTS radical-scavenging activity of the tested samples is calculated using the following formula:

ABTS scavenging activity = (Abs control − Abs sample)/Abs control × 100%,(1)

where Abs control is the absorbance of the working solution of ABTS radical in methanol and Abs sample is the absorbance of the ABTS radical working solution mixed with the sample. Trolox was used as a positive control. All determinations were performed in triplicate.

#### 2.2.2. DPPH Assay

The test for radical-scavenging activity using DPPH was carried out according to the method described by Brand-Williams et al. [48]. To the methanolic solutions of the tested compounds at different concentrations, a 1 mM methanolic solution of DPPH was added. The solutions were mixed well and left in the dark for 30 min. Absorption was measured at a wavelength of 517 nm against a control sample containing only a methanolic solution of DPPH. The percentage of DPPH radical-scavenging activity of the tested samples was calculated using the following formula:

DPPH scavenging activity = (Abs control − Abs sample)/Abs control × 100%,(2)

where Abs control is the absorbance of the working solution of DPPH radical in methanol and Abs sample is the absorbance of the DPPH radical working solution mixed with the sample. Trolox was used as a positive control. All determinations were performed in triplicate.

#### 2.2.3. FRAP Assay

The main solutions include 300 mmol/L acetate buffer with pH 3.6, 10 mmol/L TPTZ solution in 40 mmol/L HCl, and 20 mmol/L FeCl_3_.6H_2_O solution. The working solution was prepared by mixing 25 mL of acetate buffer, 2.5 mL of TPTZ solution, and 2.5 mL of FeCl_3_.6H_2_O solution, after which it was heated to 37 °C before use. To 1 mL of the tested substance solution at various concentrations, 1 mL of the FRAP solution was added, after which the samples were left in the dark for 30 min. Absorption was measured at a wavelength of 593 nm against a control sample that did not contain a solution of the tested substances. Trolox was used as a positive control. All determinations were performed in triplicate. The results are presented as Trolox Equivalent Antioxidant Capacity (TEAC).

#### 2.2.4. Antioxidant Assay Kit (CS0790) Sigma-Aldrich

The Assay Buffer, 10× was diluted ten-fold with ultrapure water and mixed well. The myoglobin was used as the source of metmyoglobin in the assay reaction. Myoglobin Stock Solution was prepared by reconstituting the Myoglobin by adding 285 µL of ultrapure water to the vial and vortexing well. Myoglobin Working Solution was prepared by diluting the Myoglobin Stock Solution 100-fold with Assay Buffer and mixing well. For each well of Trolox Standard or Test sample, 20 µL of the Myoglobin Working solution was prepared. Trolox Working Solution was prepared by reconstituting the Trolox by adding 2.67 mL of Assay Buffer and vortexing until totally dissolved. ABTS Substrate Solution was prepared by adding one ABTS tablet and one Phosphate–Citrate Buffer tablet to 100 mL of ultrapure water and mixing until totally dissolved. Trolox Standards for a standard curve were prepared by diluting Trolox working solution with Assay Buffer in different ratios. ABTS Substrate Working Solution was prepared by adding 25 µL of 3% Hydrogen Peroxide Solution to 10 mL of ABTS Substrate Solution. Assays were prepared in the 96-well plate. In wells for the Trolox standard curve, 10 µL of a Trolox Standard and 20 µL of Myoglobin Working Solution were added. In wells for the test samples, 10 µL of solutions of the tested compounds were added as well as 20 µL of Myoglobin Working Solution. A total of 150 µL of ABTS Substrate Working Solution was added to each well. The plate was incubated for 5 min at room temperature. After that, 100 µL of Stop Solution was added to each well. Prior to use, the Stop Solution was warmed to room temperature and mixed until homogeneous. The endpoint absorbance was read at 405 nm using a plate reader. The results are presented as Trolox Equivalent Antioxidant Capacity (TEAC).

### 2.3. Neuroprotection and Neurotoxicity Assays

#### 2.3.1. Animals

In total, 9 animals were used in the experiments. The animals were obtained from the National Breeding Centre of the Bulgarian Academy of Sciences, Sofia, Bulgaria and Slivnitsa, Bulgaria and kept under standard conditions in plexiglass cages with free access to water and food and 12 h/12 h light/dark regime at 20–25 °C. Twelve hours before each specific study, the animals’ food was withdrawn.

#### 2.3.2. Incubation of the Rat Brain Sub-Cellular Fractions (Synaptosomes, Mitochondria, and Microsomes)

The sub-cellular fractions were incubated with the test substances at a concentration of 100µM for 1 h—for examining their independent effect; and at concentration 50µM—pre-incubation for 30 min (only substances) and then 1 h in combination with the toxic agent.

#### 2.3.3. Preparation of Rat Brain Synaptosomes and Mitochondria

Synaptosomes and mitochondria were obtained by multiple subcellular fractionations using a Percoll gradient [49]. Brain homogenate was centrifuged at 1000× *g* for 5 min at +4 °C, and the supernatant was re-centrifuged under the same conditions; the resulting supernatants were combined, distributed into four tubes, and centrifuged at 10,000× *g* for 20 min at +4 °C three times, with the final two spins aiding purification. Isolation employed a colloidal silicon solution (Percoll): a 90% stock was prepared, followed by 16% and 10% solutions (4 mL each per tube), and the final pellet was resuspended with Percoll (7.5%) and centrifuged at 15,000× *g* for 20 min at +4 °C. This produced three layers—mitochondria at the bottom, lipids at the top, and synaptosomes at the 16–10% interface—which were collected, combined, supplemented with buffer B plus glucose, centrifuged again at 10,000× *g* for 20 min at +4 °C to exchange buffers, and finally resuspended to obtain synaptosomes and mitochondria.

#### 2.3.4. Establishing a Dopamine Model of Neurotoxicity

This in vitro model resembles the neurodegenerative processes occurring primarily in PD. The metabolism of 6-OHDA leads to the production of reactive quinones (p-quinone), which in turn lead to the formation of ROSs. Reactive metabolites and ROSs cause damage to the pre- and post-synaptic membrane and lead to neuronal cell damage [50]. Synaptosomes were incubated with 6-OHDA (150 μM) for 1 h.

#### 2.3.5. MTT Assay to Assess Synaptosomal Viability

After incubation, synaptosomes were centrifuged on a microcentrifuge for 1 min at 15,000× *g*. The pellet was mixed gently with buffer B + glucose, and the supernatant where 6-OHDA was discarded to prevent oxidation of MTT. Centrifuge again at 15,000× *g* for 1 min. After the second wash, buffer B + glucose was added to the pellet. To the ‘washed’ synaptosomes, 60 µL of MTT solution was added. The plates were incubated with the MTT solution at 37 °C for 10min. After incubation, the samples were centrifuged at 15,000× *g* for 2 min. The excess liquid was removed and a DMSO solution was used to dissolve the formed formazan crystals. After dissolution, the amount of formazan was measured spectrophotometrically at λ = 580 nm [51].

#### 2.3.6. Determination of Reduced Glutathione (GSH) in Isolated Brain Synaptosomes

After precipitation of the proteins with trichloroacetic acid, the thiol groups in the supernatant were determined by DTNB, which produced a yellow-colored compound that absorbs light at λ = 412 nm. After incubation, synaptosomes were centrifuged at 4000× *g* for 3 min. The supernatant was removed and the pellet taken for GSH determination. It was treated with 5% trichloroacetic acid, then left for 10 min on ice. Centrifuge at 8000× *g* for 10 min (2 °C). The supernatant is taken for GSH determination and frozen at −20 °C. Immediately before measurement, the samples were neutralized with 5N NaOH [52].

#### 2.3.7. *Tert*-Butyl Hydroperoxide-Induced Oxidative Stress

Isolated brain mitochondria were incubated with 75 µM *tert*-butyl hydroperoxide (t-BuOOH) [53].

#### 2.3.8. Determination of Malondialdehyde (MDA) Production in Brain Mitochondria [51]

To the mitochondria 0.3 mL of 0.2% thiobarbituric acid and 0.25 mL of sulfuric acid (0.05 M) were added, and the mixture was boiled for 30 min. After boiling, the tubes were placed on ice and 0.4 mL of n-butanol was added to each, then centrifuged at 3500× *g* for 10 min. The amount of MDA was determined spectrophotometrically at 532 nm.

#### 2.3.9. Determination of GSH Level in Brain Mitochondria [54]

After incubating the mitochondria with the substances and *tert*-butyl hydroperoxide, the reaction was stopped with 5% trichloroacetic acid, and each sample was homogenized with the acid and left on ice. After centrifugation of the homogenate at 6000× *g*, a 0.04% solution of DTNB was added to the supernatant to give a yellow color, the determination being spectrophotometric at 412 nm.

#### 2.3.10. Isolation of Brain Microsomes [55]

The brain was homogenized in 9 volume parts of 0.1 M Tris buffer containing: 0.1 mM Dithiothreitol, 0.1 mM Phenylmethylsulfonyl fluoride, 0.2 mM EDTA, 1.15% KCl, and 20% (*v*/*v*) glycerol (pH 7.4). The resulting homogenate was centrifuged twice at 17,000× *g* for 30 min. The supernatants from the two centrifugations were pooled and centrifuged twice at 100,000× *g* for 1 h. The pellet was frozen in 0.1 M Tris buffer.

#### 2.3.11. Iron/Ascorbate-Induced Lipid Peroxidation (LPO)

Non-enzyme-induced lipid peroxidation was induced with 20 μM ferrous sulfate solution and 500 μM ascorbic acid solution [56].

After incubation of the microsomes with the substances and toxic agent, the reaction was stopped by the addition of 0.5 mL of 20% trichloroacetic acid followed by 0.5 mL of 0.67% thiobarbituric acid. The ongoing reactions were associated with the formation of a colored complex between the malondialdehyde formed and thiobarbituric acid. The determination of MDA was spectrophotometric at 535 nm. A molar extinction coefficient of 1.56 × 105 M^−1^ cm^−1^ was used for the calculation.

### 2.4. Statistical Methods

The results of in vitro antioxidant tests were expressed as a mean ± SD (n = 3). Values of *p* < 0.05 were considered statistically significant. Compounds’ concentrations providing 50% radical-scavenging activity (IC_50_) were obtained by plotting the percentage inhibitions against compound concentrations. The results of the experiments performed on isolated brain synaptosomes, mitochondria, and microsomes were statistically processed using the ‘MEDCALC’ program using the non-parametric Mann–Whitney method at significance levels *p* < 0.05; *p* < 0.01 and *p* < 0.001.

### 2.5. In Silico ADME

The main physicochemical properties of the most promising representatives among the newly synthesized hydrazide–hydrazones—**VV4** and **VV6**, were predicted in silico. Molecular weight (MW), number of hydrogen bond acceptors (HBA), number of hydrogen bond donors (HBD), octanol/water partition coefficient (LogP), aqueous solubility (LogS), brain/blood partition coefficient (LogBB), and human oral absorption (HOA) were calculated using the QikProp module in Schrodinger (Schrödinger Release 2024-1: QikProp, Schrödinger, LLC, New York, NY, USA, 2024). Acid dissociation constants (pKa) and distribution coefficients at pH 7.4 (LogD) were determined by ACD Labs v. 9.08 (Swiss Institute of Bioinformatics, Vaud, Switzerland). The fraction ionized as an acid at pH = 7.4 (fA) and was calculated according to the equation fA = 1/(1 + 10^pKa−7.4^). Druglikeness of **VV4** and **VV6** was evaluated using Lipinski’s rule of five (Ro5), as implemented in the Swiss ADME software.

### 2.6. DFT Studies

A density functional theory (DFT) study of the most active compounds—**VV4** and **VV6**, was conducted using ORCA 6.1.1. The molecular structures of **VV4** and **VV6** were optimized in their ground states using the B3LYP functional with 6-311++G(d,p) basis set. Subsequently, the energies of the highest occupied molecular orbital (HOMO), the lowest unoccupied molecular orbital (LUMO) and several related electronic descriptors were calculated. These are the energy gaps (ΔE) between LUMO and HOMO energies, ionization potential (IP = −EHOMO), electron affinity (A = −ELUMO), chemical potential (µ = −(IP + A)/2), hardness (η = (IP − A)/2), Mulliken electronegativity (χ = (IP + A)/2), softness (S = 1/2η), electrophilicity index (ω = µ2/2η), and maximum charge transfer (∆Nmax = (IP + A)/2(IP − A)). Finally, frontier molecular orbital (FMO) analyses were performed. The DFT calculations were rerun using the M06-2X functional with the 6-311++G(d,p) basis set in Jaguar, as implemented in Maestro (Schrödinger Release 2026-2: Jaguar, Schrödinger, LLC, New York, NY, USA, 2025). The geometries of the selected compounds were optimized and the corresponding electronic descriptors were obtained from the converged ground-state structures.

## 3. Results

### 3.1. Synthesis

**Scheme 1:**
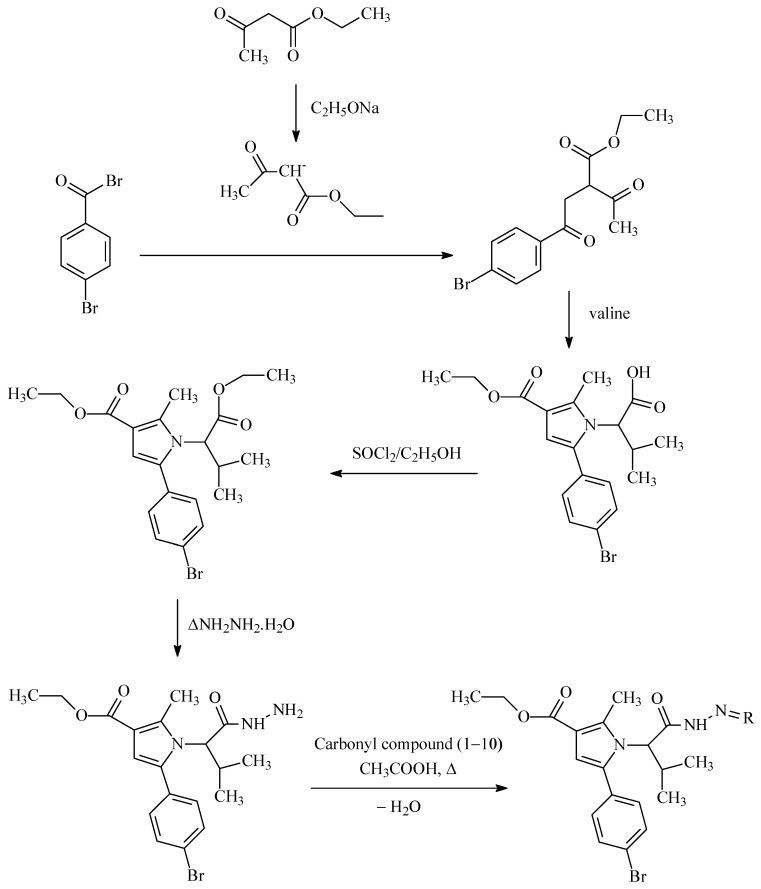
Synthetic scheme for obtaining the target pyrrole derivatives.

The target compounds were synthesized via a multistep synthetic route outlined in Scheme 1. Initially, a 1,4-dicarbonyl intermediate was prepared and subsequently subjected to Paal–Knorr condensation with the amino acid valine, affording the corresponding N-pyrrolylcarboxylic acid derivative. This intermediate then underwent esterification, followed by hydrazinolysis of the resulting ester to yield the target hydrazide. Finally, azomethine condensation of the hydrazide with various carbonyl compounds furnished the desired hydrazide–hydrazone derivatives.

**Scheme 2:**
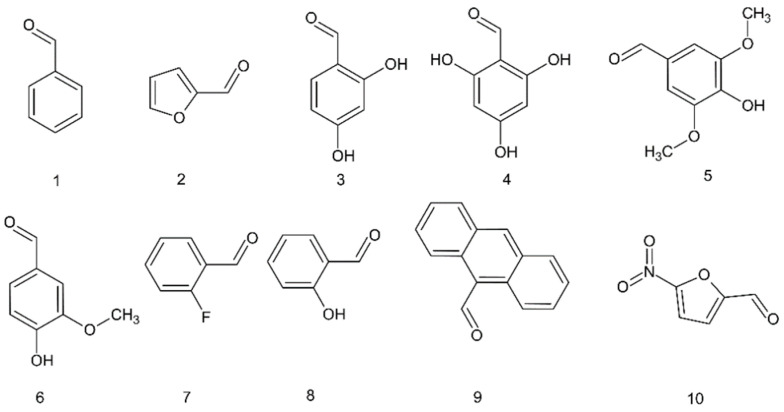
Carbonyl compounds used in the synthesis of target hydrazide–hydrazones.

The carbonyl compounds used for the synthesis of hydrazide–hydrazones are shown in Scheme 2.

### 3.2. Antioxidant Assays

#### 3.2.1. ABTS Test

Using the algorithm described in Section 2.2.1., an ABTS test for radical-scavenging activity of the newly synthesized pyrrole-containing compounds was conducted. The results of it are presented in Table 1.

The obtained results indicate that three of the newly synthesized compounds, namely **VV3**, **VV4**, and **VV6**, exhibited ABTS radical-scavenging activity exceeding that of the reference standard Trolox. In contrast, compounds **VV2**, **VV5**, and **VV7** demonstrated considerably lower antioxidant activity, whereas compounds **VV1**, **VV8**, **VV9**, and **VV10** showed no detectable ABTS radical-scavenging activity under the applied experimental conditions.

#### 3.2.2. DPPH Test

Using the algorithm described in Section 2.2.2., a DPPH test for antioxidant activity of the newly synthesized pyrrole derivatives was performed. The results are presented in Table 2.

The obtained results demonstrated that four compounds, namely **VV3**, **VV4**, **VV5**, and **VV6**, exhibited DPPH radical-scavenging activity. Among them, compounds **VV4** and **VV6** showed higher antioxidant activity than the reference standard Trolox, whereas **VV3** displayed slightly lower activity. In contrast, **VV5** exhibited markedly reduced radical-scavenging capacity. The remaining six compounds showed no detectable DPPH radical-scavenging activity under the experimental conditions employed.

#### 3.2.3. FRAP Test

Using the algorithm described in Section 2.2.3., a FRAP test for antioxidant activity of the newly synthesized pyrrole derivatives was performed. The results are presented in Table 3.

The indicated results show that again compounds **VV4** and **VV6** possess the most pronounced activity, with **VV6** showing the highest. Compound **VV3** also demonstrates higher activity than the standard Trolox. In the remaining seven compounds, the antioxidant activity is significantly lower or absent.

#### 3.2.4. Antioxidant Assay Kit (CS0790) Sigma-Aldrich

Using the algorithm described in Section 2.2.4., an antioxidant test of the newly synthesized pyrrole derivatives was performed. The results are presented in Table 4.

As with the other antioxidant tests, compound **VV6** demonstrated the highest activity. The other compounds with stronger activity than the standard Trolox are **VV4** and **VV3**, while the remaining seven compounds showed low or no antioxidant activity.

### 3.3. Neurotoxicity Evaluations

#### 3.3.1. Effect of Substances on Biomarkers, Characterizing the Functional–Metabolic Profile of Brain Synaptosomes

**Figure 1:**
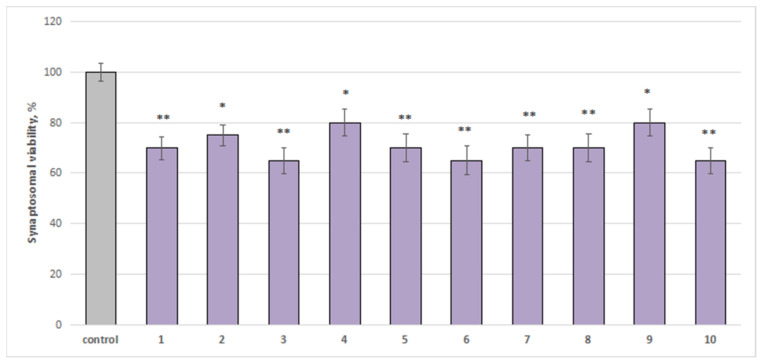
Effect of the test substances applied alone at a concentration of 100 µM on synaptosomal viability. * *p* < 0.05; ** *p* < 0.01 vs. control (non-treated synaptosomes).

**Figure 2:**
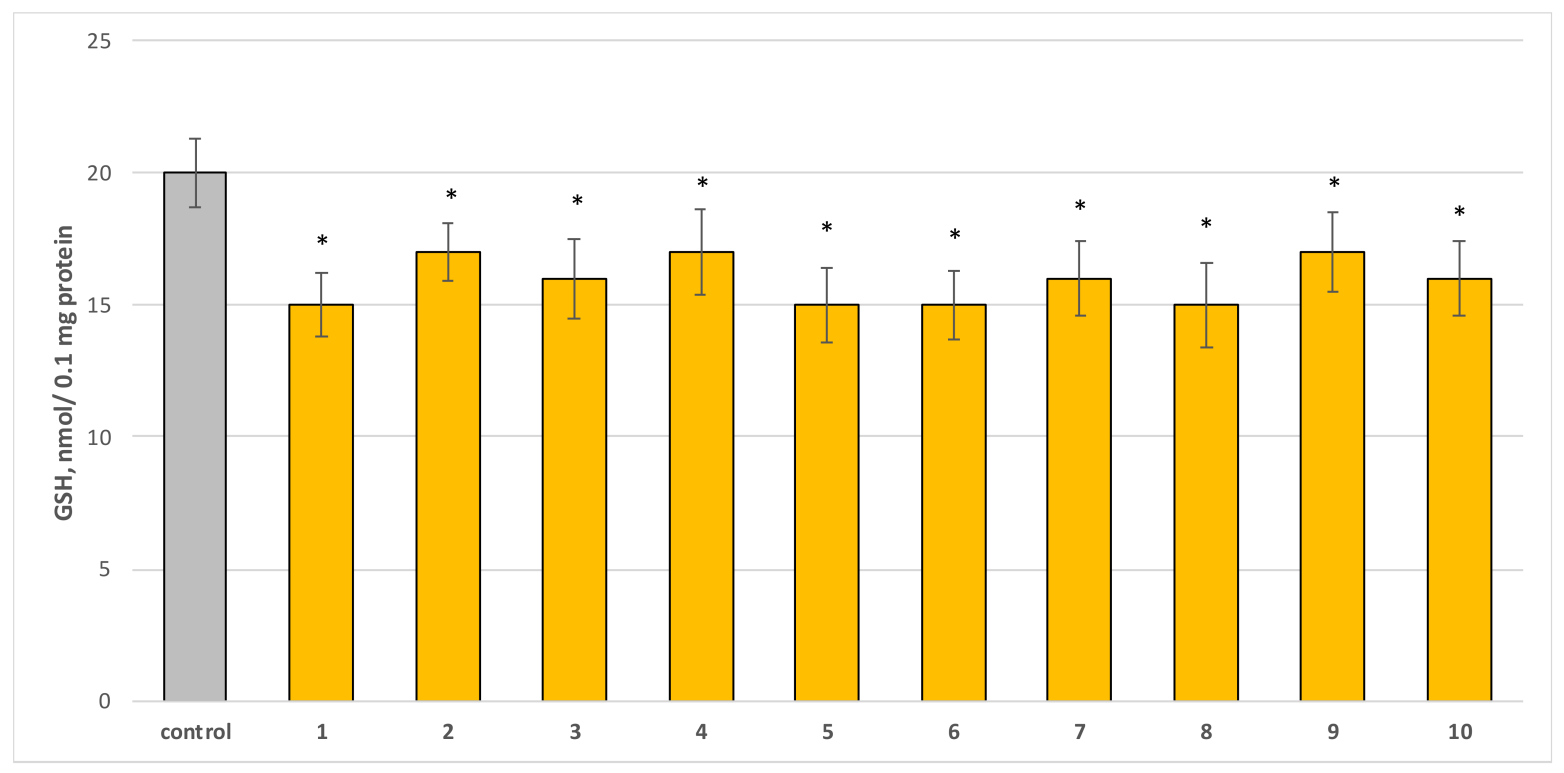
Effect of the test substances applied alone at a concentration of 100 µM on the level of GSH. * *p* < 0.05 vs. control (non-treated synaptosomes).

When administered alone, at a concentration of 100 µM, to brain synaptosomes, the test substances (**VV1**–**VV10**) exhibited a statistically significant weak neurotoxic effect compared to the control (untreated synaptosomes). They slightly decreased synaptosomal viability and reduced glutathione level (Figure 1 and Figure 2).

Substances **VV1**, **VV5**, **VV7**, and **VV8** statistically significantly decreased synaptosomal viability by 30%; **VV3**, **VV6**, and **VV10**—decreased by 35%; **VV2**—by 25% and **VV4** and **VV9**—by 20%, compared to the control.

Substances **VV1**, **VV5**, **VV6**, and **VV8** statistically significantly decreased the GSH level by 25%; **VV3**, **VV7**, and **VV10**—decreased by 20%, and **VV2**, **VV4**, **VV9**—by 15%, compared to the control.

#### 3.3.2. Effect of Substances, Administered Alone, on Biomarkers Characterizing the Functional–Metabolic Profile of Brain Mitochondria

**Figure 3:**
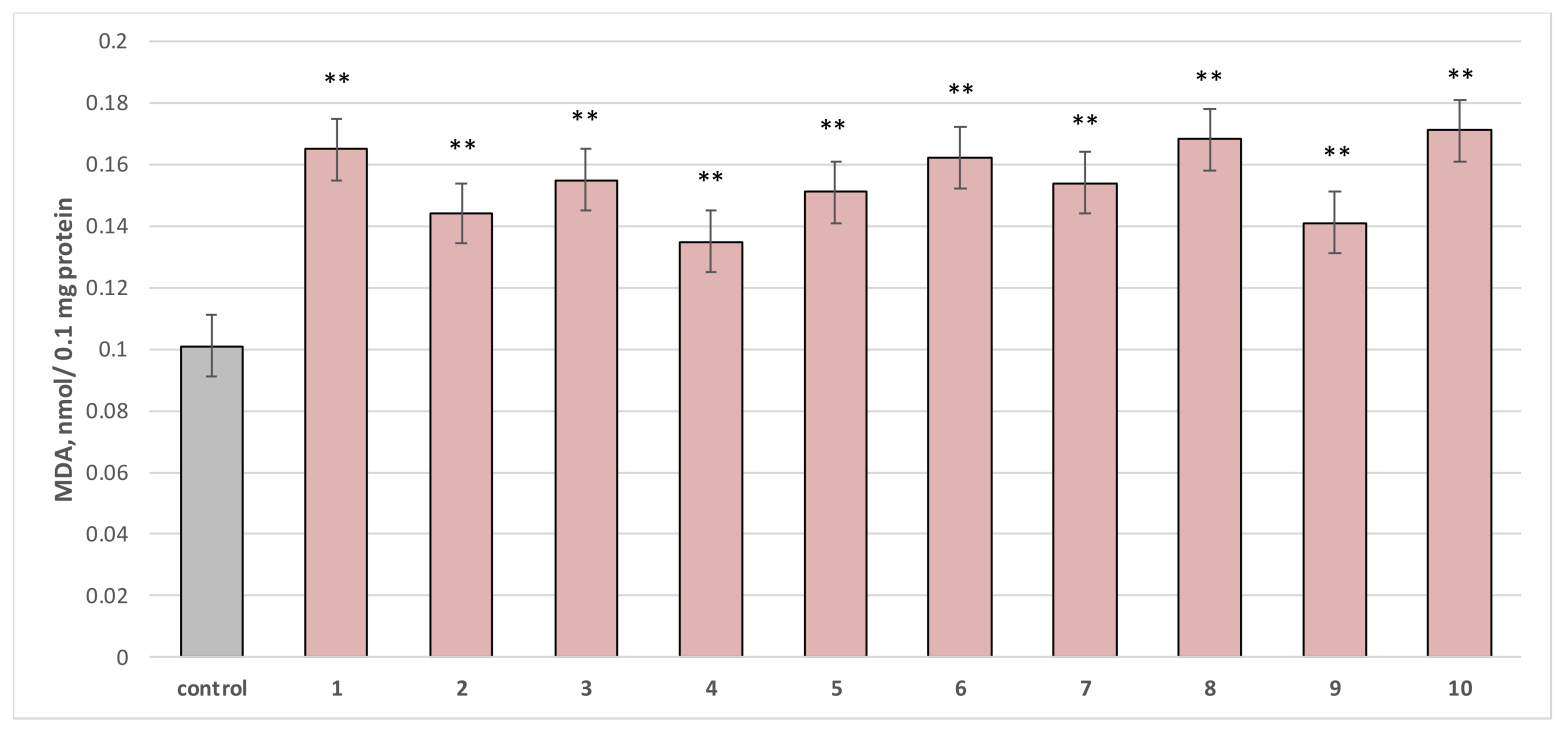
Effect of test substances applied alone at a concentration of 100 µM on MDA production. ** *p* < 0.01 vs. control (non-treated mitochondria).

**Figure 4:**
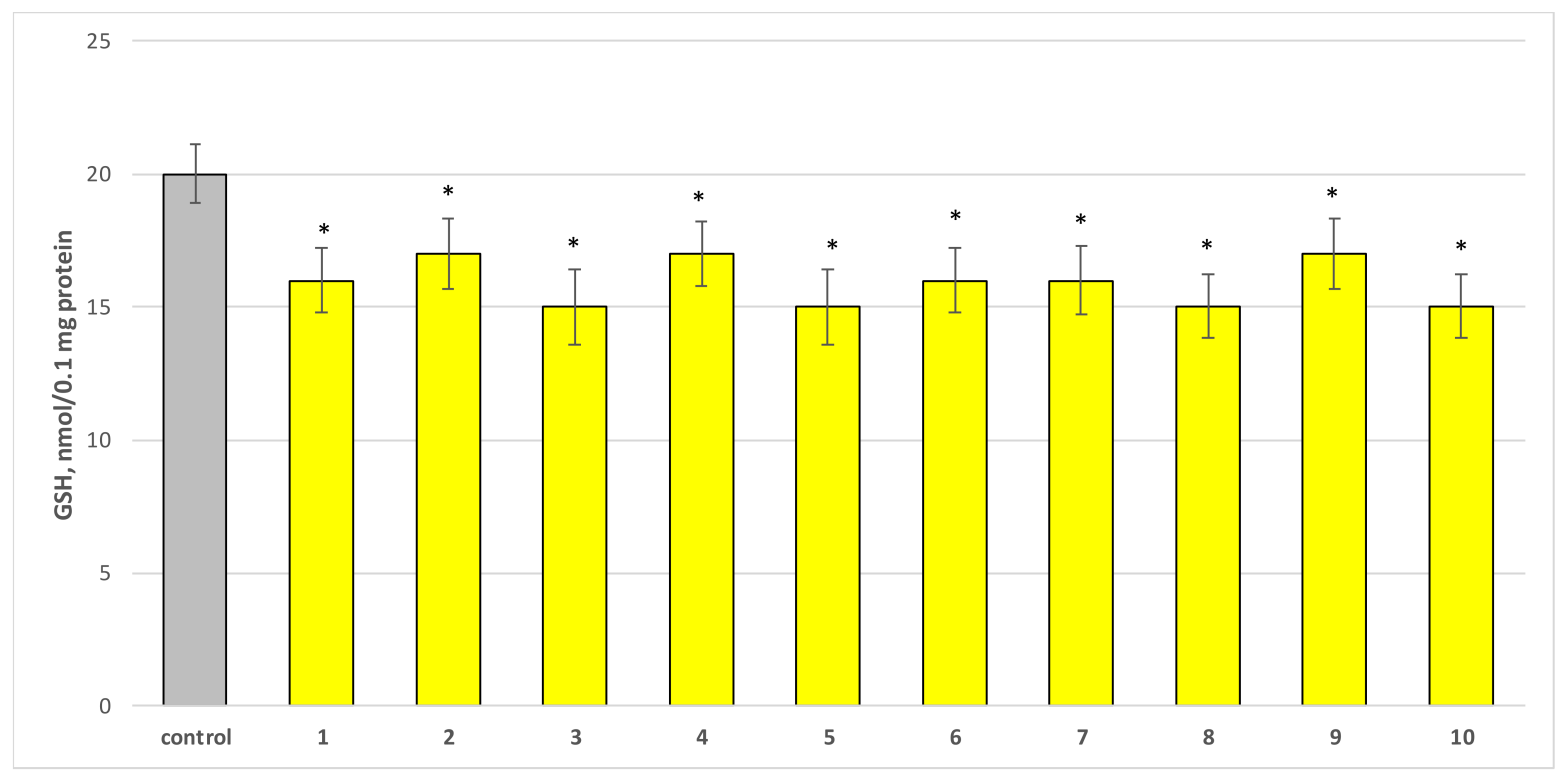
Effect of test substances applied alone at a concentration of 100 µM on GSH level. * *p* < 0.05 vs. control (non-treated mitochondria).

When applied alone, at a concentration of 100 µM, to brain mitochondria, the test substances (**VV1**–**VV10**) again exhibited a statistically significant weak neurotoxic effect, relative to the control (untreated mitochondria). They slightly increased malondialdehyde (MDA) production and decreased reduced glutathione (GSH) level (Figure 3 and Figure 4).

**VV1** increased MDA production statistically significantly by 63%; **VV2**—increased by 43%; **VV3**—by 53%; **VV4**—by 34%; **VV5**—by 50%; **VV6**—by 60%; **VV7**—by 52%; **VV8**—by 66%; **VV9**—by 40%; and **VV10**—by 69%, compared to the control.

**VV1**, **VV6,** and **VV7** decreased GSH level statistically significantly by 20%; **VV2**, **VV4**, and **VV9**—decreased by 15%; **VV3**, **VV5**, **VV8**, and **VV10**—by 25%, compared to the control.

#### 3.3.3. Effect of Substances, Administered Alone, on Biomarkers Characterizing the Functional–Metabolic Profile of Brain Microsomes

**Figure 5:**
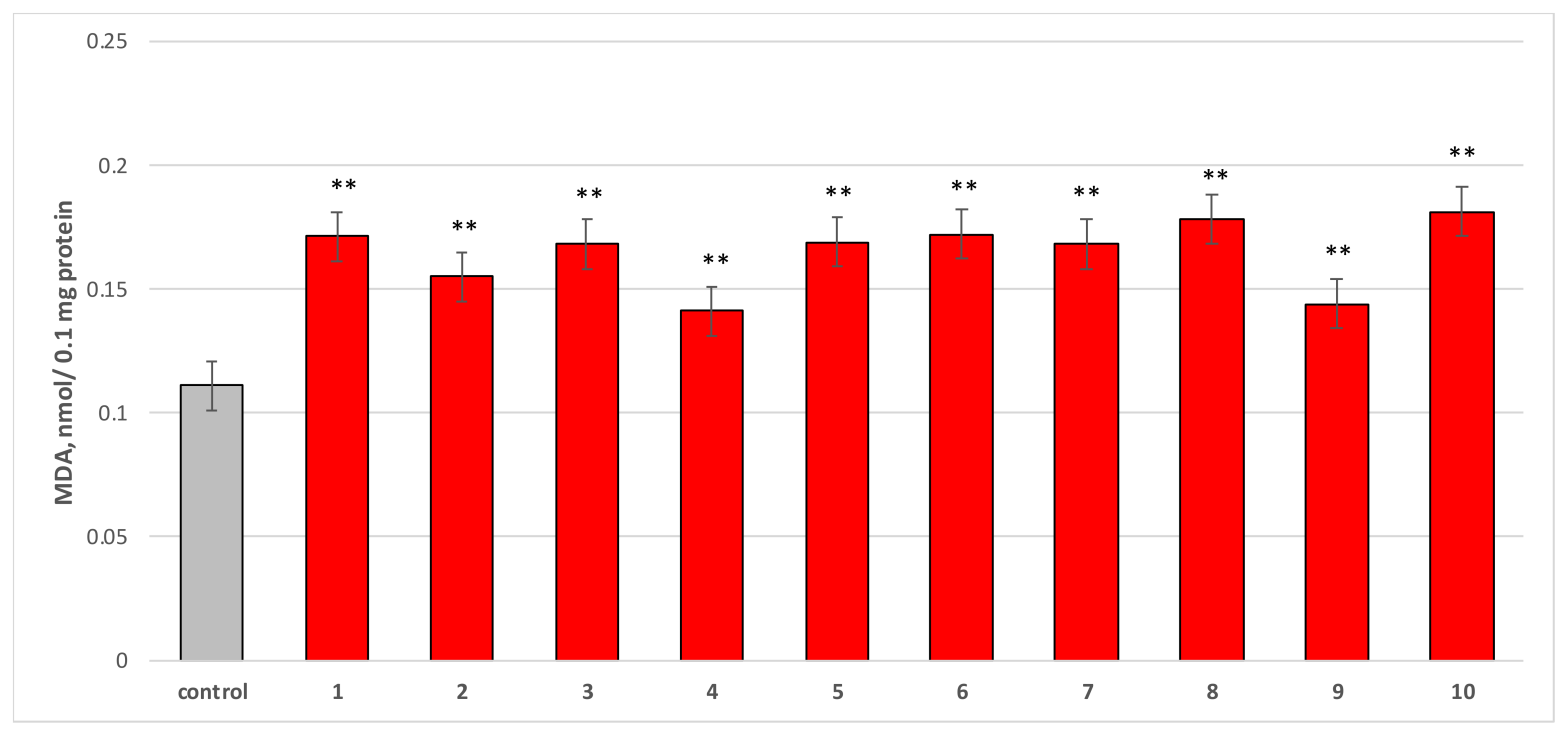
Effect of test substances applied alone, at a concentration of 100 µM, on MDA production. ** *p* < 0.01 vs. control (non-treated microsomes).

Self-administered, at a concentration of 100 µM, to brain microsomes, the test substances (**VV1**–**VV10**) again exhibited a statistically significant weak neurotoxic effect, relative to the control (untreated microsomes). They slightly increased the production of malondialdehyde (MDA), a marker of lipid peroxidation (Figure 5).

**VV1** increased MDA production statistically significantly by 54%; **VV2**—increased by 40%; **VV3**—by 51%; **VV4**—by 27%; **VV5**—by 52%; **VV6**—by 55%; **VV7**—by 51%; **VV8**—by 60%; **VV9**—by 30%; and **VV10**—by 63%, compared to the control.

### 3.4. Neuroprotection Evaluations

#### 3.4.1. Effect of Substances in a Model of 6-OHDA-Induced Neurotoxicity on Parameters Characterizing the Functional–Metabolic Profile of Brain Synaptosomes

**Figure 6:**
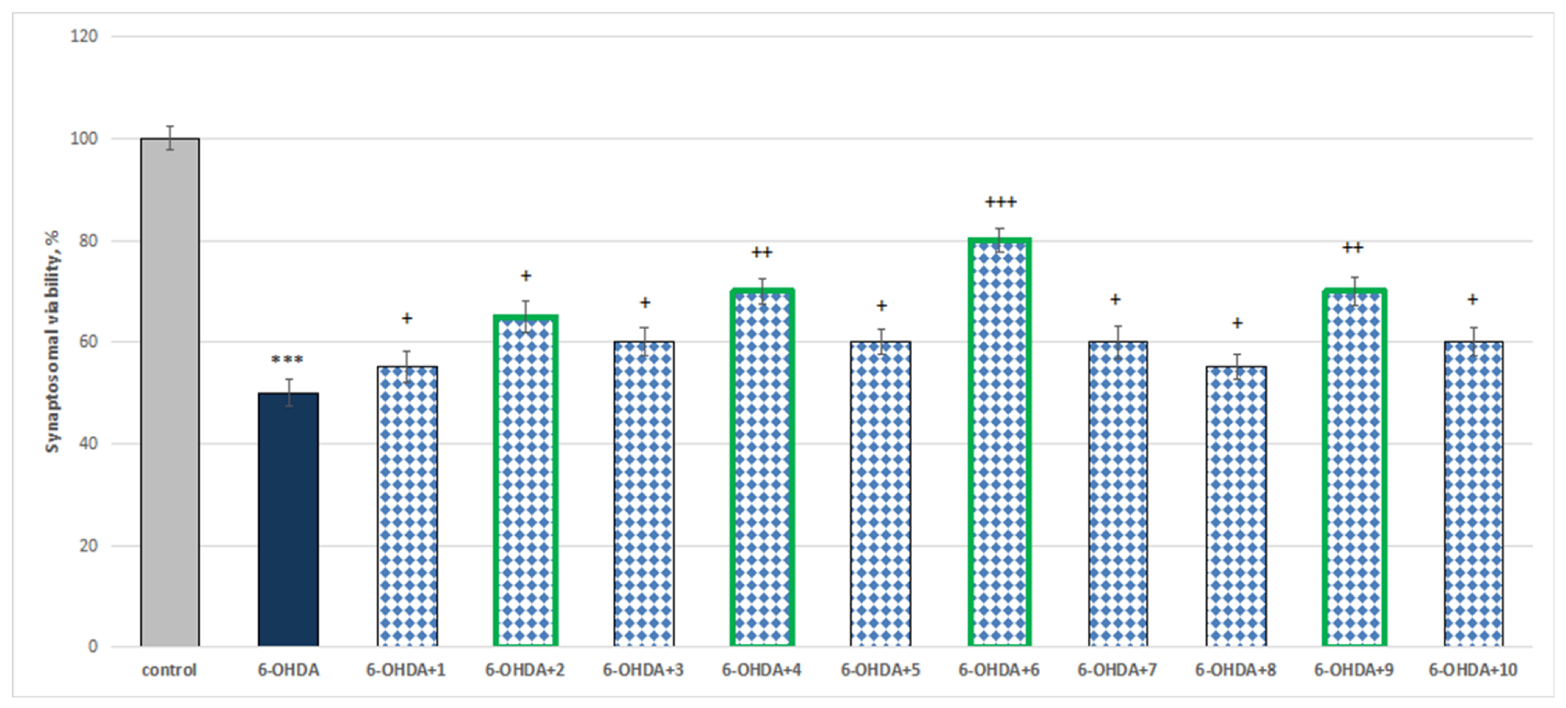
Effect of substances, in combination with 6-OHDA, on synaptosomal viability. *** *p* < 0.001 vs. control (non-treated synaptosomes); + *p* < 0.05; ++ *p* < 0.01; +++ *p* < 0.01 vs. 6-OHDA.

**Figure 7:**
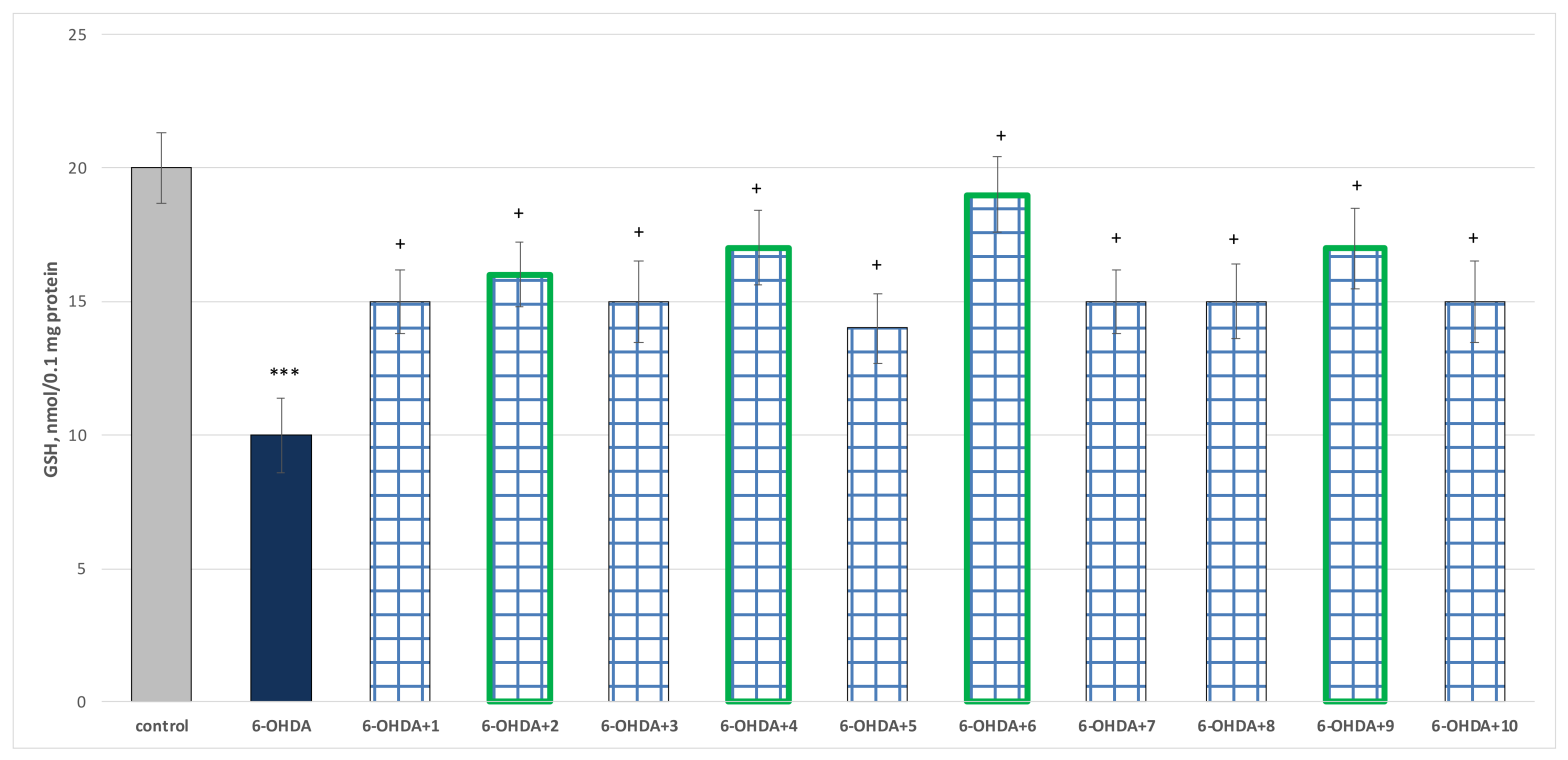
Effect of substances, in combination with 6-OHDA, on the level of reduced glutathione (GSH). *** *p* < 0.001 vs. control (non-treated synaptosomes); + *p* < 0.05 vs. 6-OHDA.

The toxic agent (6-OHDA), applied alone at a concentration of 150 µM, to isolated synaptosomes resulted in a statistically significant 50% decrease in synaptosomal viability and GSH level compared to the control (untreated synaptosomes) (Figure 6 and Figure 7).

Substances **VV1** and **VV8** statistically significantly preserved synaptosomal viability by 10%; **VV3**, **VV5**, **VV7**, and **VV10**—preserved by 20%; **VV2**—by 30%, **VV6**—by 60%, and **VV4** and **VV9**—by 40%, compared to the toxic agent (6-OHDA).

Substances **VV1**, **VV3**, **VV7**, **VV8**, and **VV10** statistically significantly preserved GSH level by 50%; **VV5**—preserved by 40%; **VV2**, **VV4**, **VV9**—by 60%, and **VV6**—by 70%, compared to the toxic agent (6-OHDA).

When combined with 6-OHDA, all substances (at a concentration of 50 µM) exhibited a statistically significant neuroprotective effect against the toxic agent. However, the substances **VV2**, **VV4**, **VV6**, and **VV9** showed the most pronounced neuroprotection on the studied parameters (Figure 6 and Figure 7).

#### 3.4.2. Effect of Substances in a Model of t-BuOOH-Induced Oxidative Stress on Parameters Characterizing the Functional–Metabolic Profile of Brain Mitochondria

**Figure 8:**
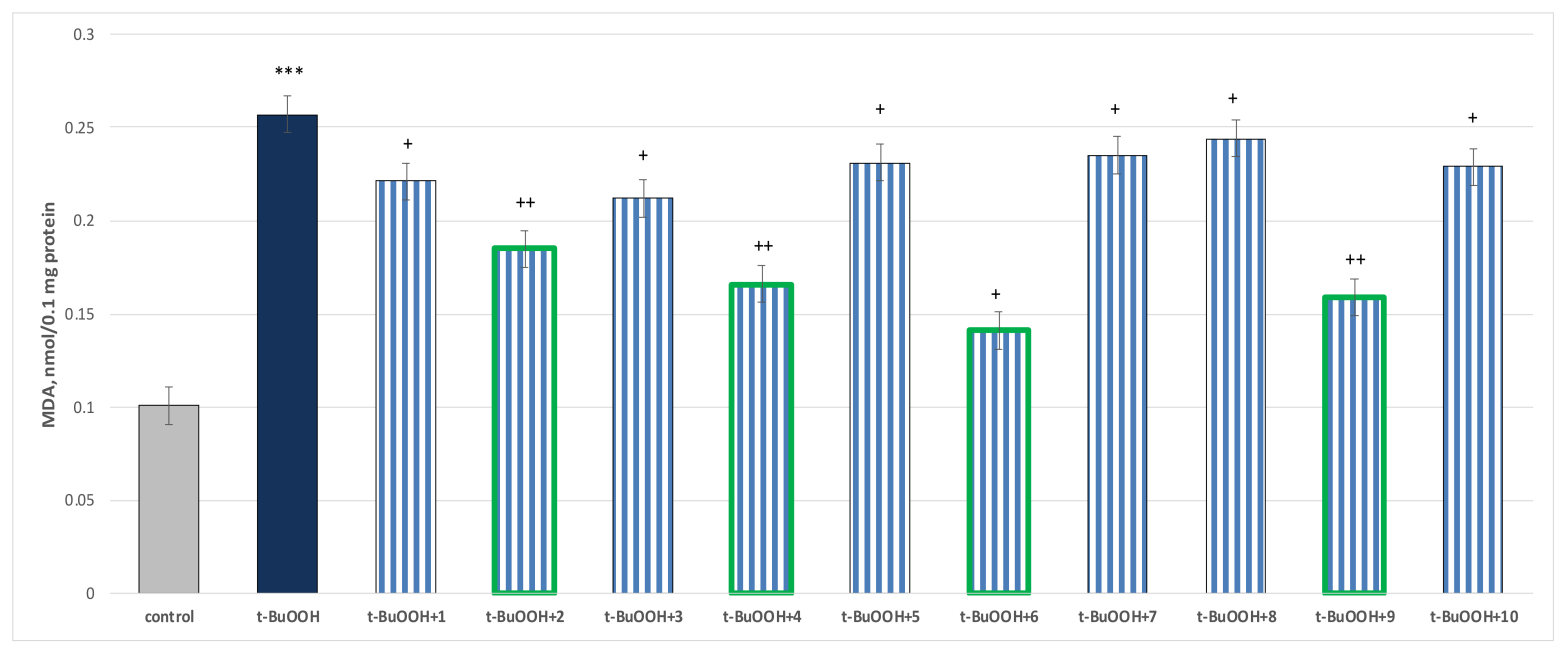
Effect of substances, in combination with *t*-BuOOH, on MDA production. *** *p* < 0.001 vs. control (non-treated mitochondria); + *p* < 0.05; ++ *p* < 0.01 vs. *t*-BuOOH.

**Figure 9:**
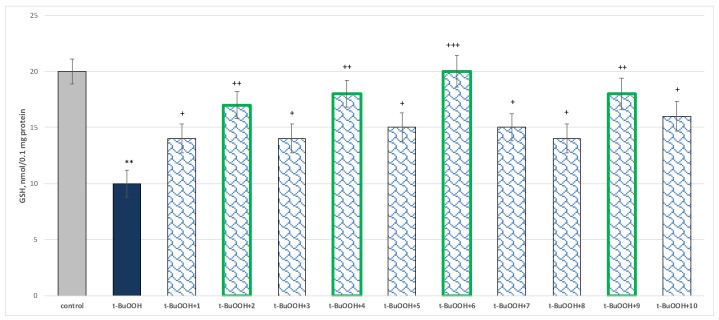
Effect of substances, in combination with *t*-BuOOH, on GSH level. ** *p* < 0.01 vs. control (non-treated mitochondria); + *p* < 0.05; ++ *p* < 0.01; +++ *p* < 0.01 vs. *t*-BuOOH.

In isolated brain mitochondria, *t*-BuOOH applied alone statistically significantly increased MDA production by 154% and decreased GSH level by 50%, compared to control (untreated mitochondria) (Figure 8 and Figure 9).

**VV1** reduced MDA production statistically significantly by 14%; **VV2**—reduced by 28%; **VV3**—by 18%; **VV4**—by 35%; **VV5**—by 10%; **VV6**—by 45%; **VV7**—by 9%; **VV8**—by 5%; **VV9**—by 38%; and **VV10**—by 11%, compared to the toxic agent (*t*-BuOOH).

**VV1**, **VV3**, and **VV8** preserved GSH level statistically significantly by 40%; **VV2**—preserved by 70%; **VV4** and **VV9**—by 80%; **VV5** and **VV7**—by 50%; **VV6**—by 100% and **VV10**—with 60%, compared to the toxic agent (*t*-BuOOH).

When combined with *t*-BuOOH, again all substances (at a concentration of 50 µM) exhibited a statistically significant neuroprotective effect, compared to the toxic agent. The substances **VV2**, **VV4**, **VV6**, and **VV9** again showed the most pronounced neuroprotection on the parameters studied (Figure 8 and Figure 9).

The protective effects are likely due, on the one hand, to the preservation of reduced glutathione levels and, on the other hand, to the scavenging of free radicals generated during *t*-BuOOH metabolism.3.4.3. Effect of substances in a model of iron/ascorbate-induced lipid peroxidation (LPO) on parameters characterizing the functional–metabolic profile of brain mitochondria.

**Figure 10:**
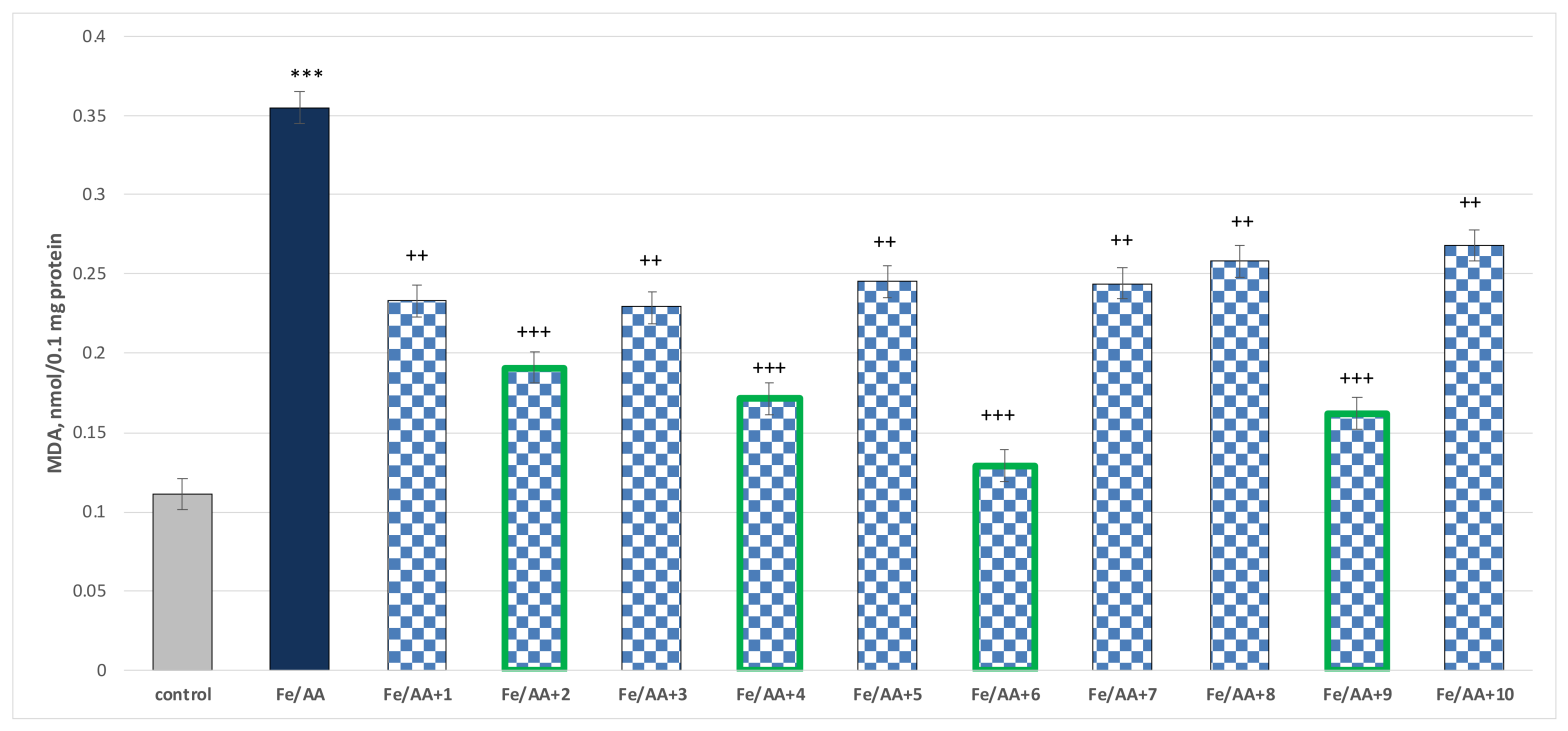
Effect of substances under conditions of non-enzyme-induced lipid peroxidation (Fe^2+^/AA). *** *p* < 0.001 vs. control (non-treated microsomes); ++ *p* < 0.05; +++ *p* < 0.05 vs. Fe^2+^/AA.

Incubation of brain microsomes with Fe^2+^/AA resulted in a statistically significant 220% increase in MDA production relative to control (untreated microsomes) (Figure 10).

**VV1** decreased MDA production statistically significantly by 34%; **VV2**—decreased by 46%; **VV3**—by 35%; **VV4**—by 52%; **VV5**—by 31%; **VV6**—by 64%; **VV7**—by 31%; **VV8**—by 27%; **VV9**—by 54%; and **VV10**—by 25%, compared to the toxic agent (Fe^2+^/AA).

In combination with Fe^2+^/AA, again all substances (at a concentration of 50 µM) exhibited a statistically significant antioxidant effect against the toxic agent. The substances **VV2**, **VV4**, **VV6**, and **VV9** again had the most prominent effect on MDA production (Figure 10).

Two of the investigated compounds, **VV4** and **VV6**, showed the most favorable performance in both antioxidant activity assays and neuroprotection and neurotoxicity evaluations. They were further subjected to in silico ADME analysis and DFT studies to assess their drug-like potential and to gain insight into the molecular characteristics responsible for observed effects.

### 3.5. In Silico ADME

In silico calculated physicochemical parameters of the most promising novel compounds—**VV4** and **VV6**, are presented in Table 5. The results show that both compounds satisfy the requirements of Lipinski’s Ro5 with the only exception of molecular weight. They are weak acids but differ in their degree of ionization at physiological pH. As indicated by the **f_A_** parameter, **VV4** is approximately half ionized, whereas **VV6** is predominantly in its neutral form. A comparison of **LogP** and **LogD** values suggests that the distribution of **VV6** is independent of pH, which is consistent with its higher predicted oral absorption (**HOA**) and more favorable brain/blood partitioning (**LogBB**). Both compounds exhibit poor water solubility in their neutral form.

### 3.6. DFT Studies

**Figure 11:**
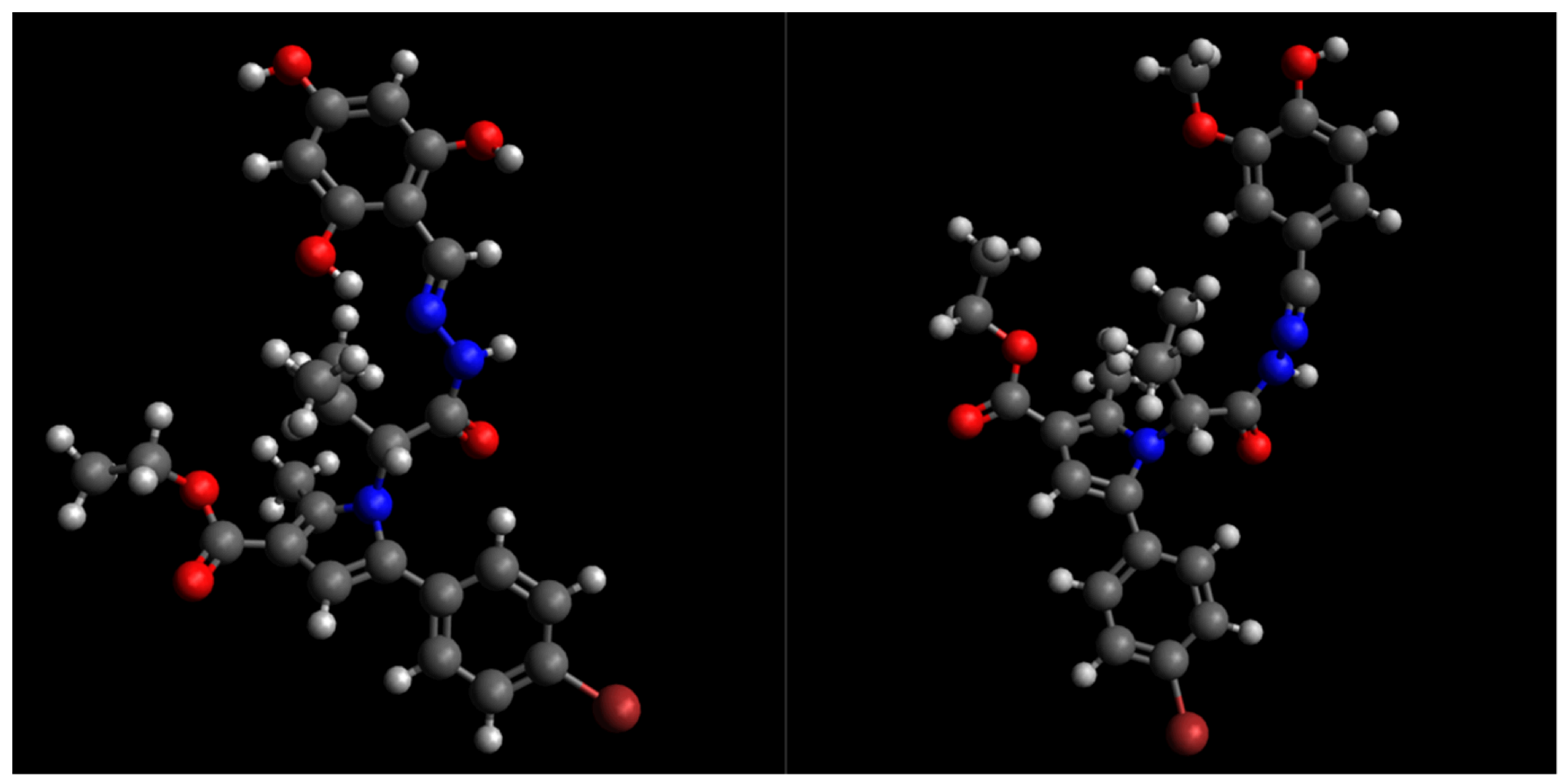
Optimized structures of compounds **VV4** (**left**) and **VV6** (**right**).

The DFT-optimized structures of **VV4** and **VV6** are shown in Figure 11.

For a more complete clarification of the electronic nature and mechanisms of action of compounds, HOMO and LUMO energies, along with the key global reactivity descriptors, were calculated. The corresponding results are summarized in Table 6. The electronic parameters of both compounds are broadly comparable. Differences in magnitude, exceeding the lower limit of the DFT method uncertainty (0.1 eV), are observed in descriptors associated mainly with the reactivity and electron-donating properties. These descriptors are the HOMO energy, HOMO–LUMO energy gap (ΔE), ionization potential (IP), and electrophilicity index (ω). Although the differences are modest, they are considered significant because the calculations are performed on congeneric compounds using the same computational method and basis set, ensuring a consistent and reliable comparison. Compared to **VV4**, **VV6** displays a higher HOMO energy, a smaller ΔE, a lower IP and a higher ω value.

**Figure 12:**
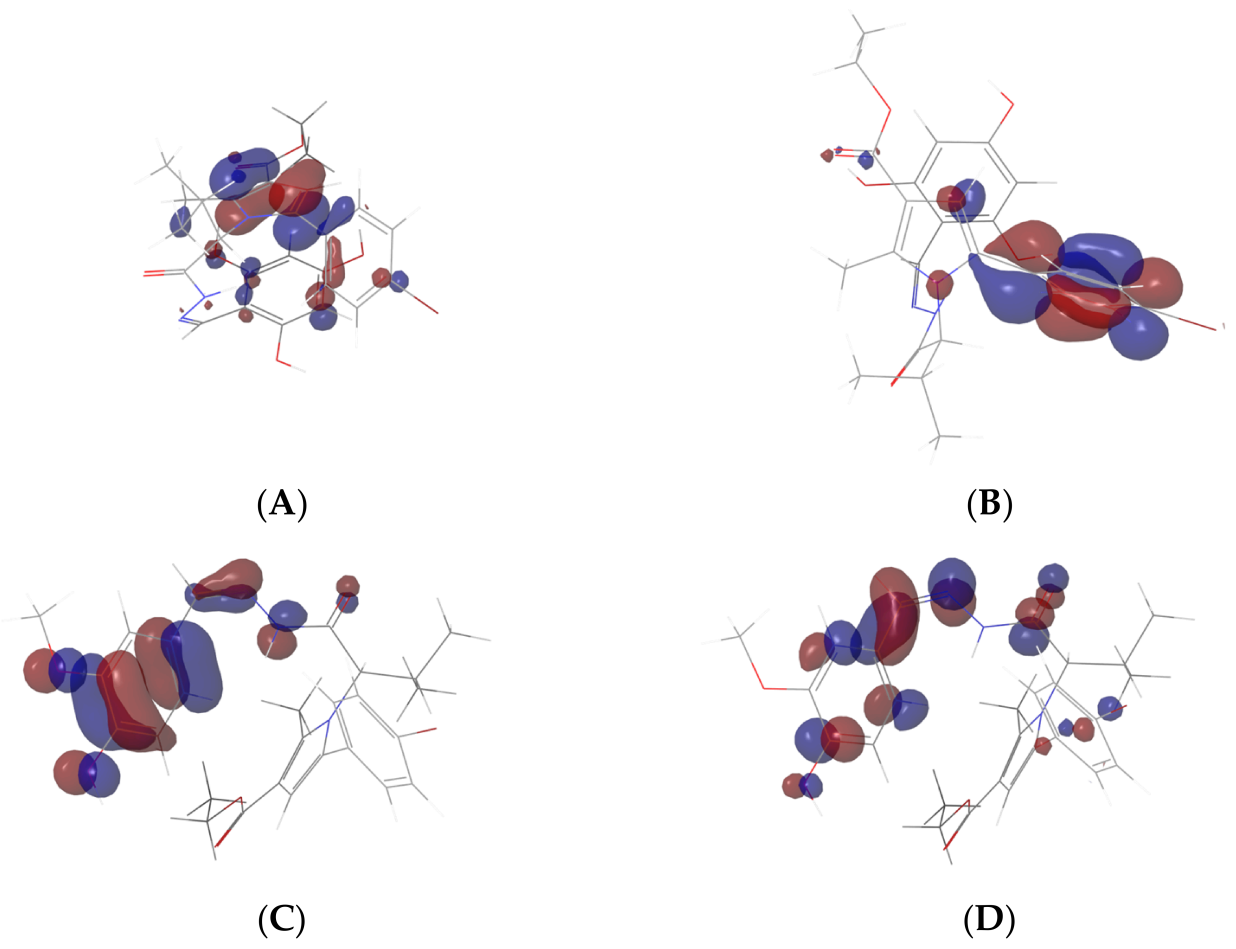
HOMO and LUMO isosurfaces calculated using Jaguar (Schrödinger) with the M06-2X functional and the 6-311++G(d,p) basis set. Panel (**A**) shows the HOMO of **VV4** and panel (**C**) shows the HOMO of **VV6**, respectively. Panel (**B**) shows the LUMO of **VV4** and panel (**D**) shows the LUMO of **VV6**, respectively.

The DFT calculations were rerun using the M06-2X functional with the 6-311++G(d,p) basis set in Jaguar, as implemented in Maestro (Schrödinger Release 2026-2: Jaguar, Schrödinger, LLC, New York, NY, USA, 2025). The geometries of the selected compounds were optimized, and the corresponding electronic descriptors were obtained from the converged ground-state structures. For **VV4**, the HOMO and LUMO energies were −7.07 eV and −1.17 eV, respectively, giving an energy gap of 5.90 eV (Figure 12A,C). For **VV6**, the HOMO and LUMO energies were −7.06 eV and −1.21 eV, respectively, with an energy gap of 5.85 eV (Figure 12B,D).

**Figure 13:**
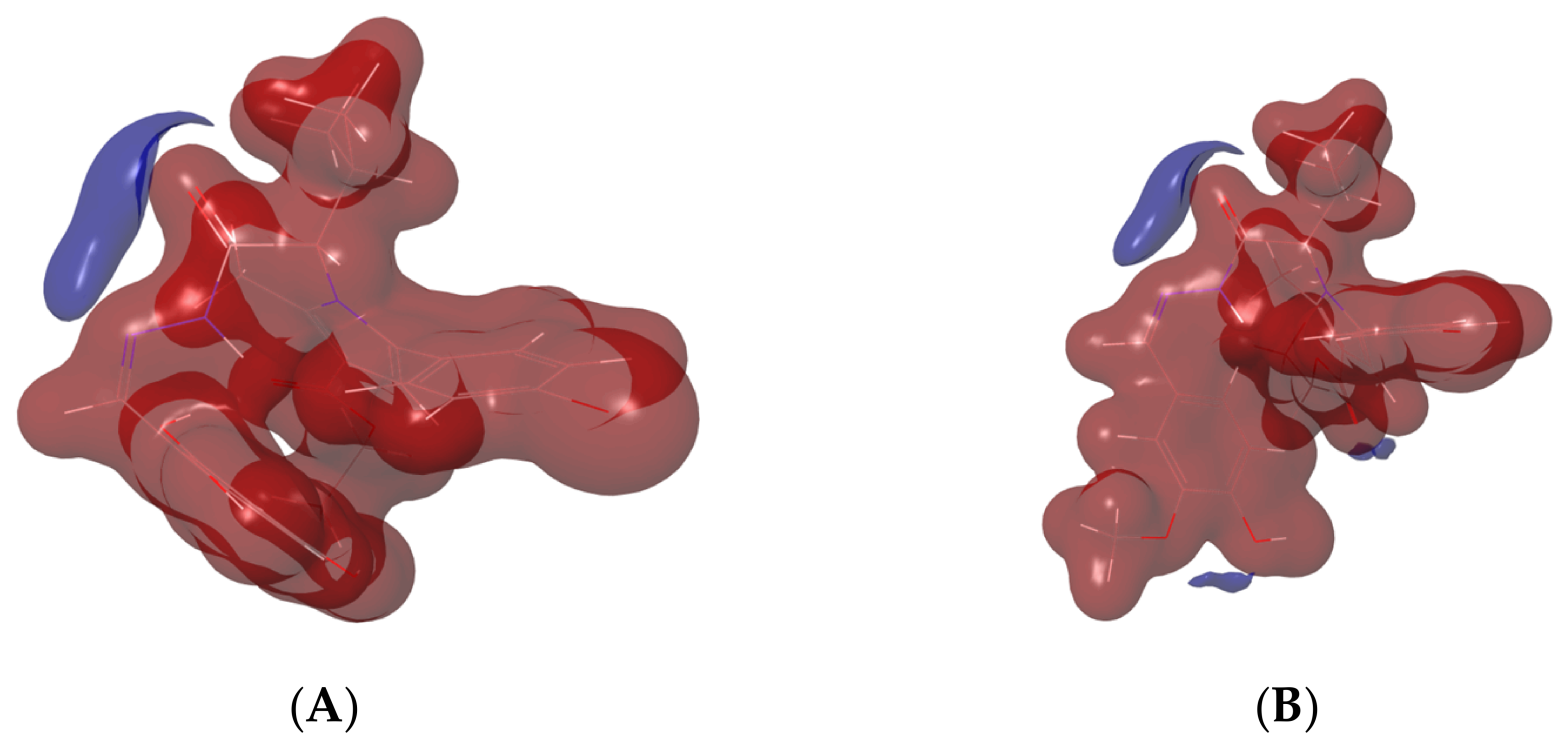
Molecular electrostatic potential surfaces of **VV4** (**A**) and **VV6** (**B**) calculated using Jaguar (Schrödinger) with the M06-2X functional and the 6-311++G(d,p) basis set. Red regions indicate negative electrostatic potential, while blue regions indicate positive electrostatic potential. Panel (**A**) shows **VV4** and panel (**B**) shows **VV6**, respectively.

The molecular electrostatic potential surfaces of **VV4** and **VV6** revealed pronounced regions of negative potential around the oxygen atoms of the hydroxyl groups and positive potential over the hydrogen-bearing regions (Figure 13). This distribution indicates that the phenolic hydroxyl groups are the most likely sites for electrophilic/radical interactions and supports their role in antioxidant activity. In both compounds, the negative potential was more extensive in the hydroxyl-substituted aromatic regions, consistent with easier electron donation and radical stabilization. These findings complement the HOMO/LUMO analysis and suggest that the hydroxyl-rich substituents contribute to the superior antioxidant behavior of **VV4** and **VV6**.

## 4. Discussion

### 4.1. Synthesis Strategy

The synthetic strategy employed in the present study constitutes a rational and efficient approach for the preparation of novel pyrrole-based hydrazide–hydrazone derivatives, combining well-established transformations with a high degree of mechanistic and chemoselective control. The key 1,4-dicarbonyl intermediate, required for subsequent Paal–Knorr cyclization, was synthesized via C-alkylation of ethyl acetoacetate with 2,4′-dibromoacetophenone in the presence of sodium ethoxide. This transformation proceeds through the generation of a resonance-stabilized enolate intermediate, which undergoes nucleophilic substitution under mild anhydrous conditions. Careful exclusion of moisture was essential to suppress competing O-alkylation processes and to favor selective C-alkylation, thereby improving the efficiency of the reaction.

Construction of the pyrrole scaffold through Paal–Knorr condensation with valine further demonstrates the versatility and robustness of this classical heterocyclization methodology. Incorporation of an α-amino acid residue introduces additional structural and physicochemical diversity, which may be advantageous for the modulation of biological activity. In the context of antioxidant agents, amino acid-derived substituents can contribute to enhanced solubility, improved pharmacokinetic behavior, and altered radical-scavenging potential. Moreover, the operational simplicity and broad substrate tolerance of the Paal–Knorr reaction render this approach highly suitable for the generation of structurally diverse compound libraries.

Transformation of the resulting N-pyrrolylcarboxylic acid into the corresponding ester constituted a key activation step for further derivatization. This process was achieved through in situ formation of the acid chloride using thionyl chloride, followed by ethanolysis, providing the ester intermediate in a synthetically accessible and efficient manner. The high reactivity of the acid chloride intermediate facilitated smooth progression to subsequent functionalization steps.

An important aspect of the synthetic sequence is the pronounced chemoselectivity observed during hydrazinolysis of the diester intermediate. The selective formation of the mono-hydrazide can be rationalized by the distinct electronic environments of the two ester functionalities. Conjugation of the ester group adjacent to the pyrrole ring decreases its electrophilic character through resonance stabilization, thereby reducing its susceptibility toward nucleophilic attack. In contrast, the ester moiety associated with the valine fragment remains comparatively more electrophilic and, consequently, reacts preferentially with hydrazine hydrate. This selective transformation enabled efficient access to the desired hydrazide intermediate, which served as a versatile precursor for subsequent hydrazone formation. This selectivity is essential for obtaining structurally defined intermediates and is consistent with previously reported findings [46].

The final step involves condensation of the synthesized hydrazide with various carbonyl compounds to afford hydrazide–hydrazones via azomethine formation. This transformation proceeds through nucleophilic addition followed by dehydration, forming the characteristic C=N bond. The use of glacial acetic acid as both solvent and catalyst promotes reaction efficiency and product stability.

Hydrazide–hydrazone derivatives are recognized for their broad spectrum of biological activities, including pronounced antioxidant potential, which is frequently associated with the presence of the azomethine (–C=N–) functionality and the extended conjugated framework. These structural features facilitate electron delocalization and may promote efficient stabilization of radical species formed during redox processes. Furthermore, incorporation of the pyrrole nucleus is expected to enhance π-electron conjugation, thereby increasing the ability of the molecules to stabilize reactive intermediates through resonance effects. Such electronic characteristics may contribute significantly to the radical-scavenging capacity and overall redox activity of the synthesized compounds.

The described methodology aligns with established strategies for pyrrole and hydrazone synthesis but offers improvements in selectivity and functional group compatibility. The selective hydrazinolysis step, in particular, represents a notable advantage, as it avoids overreaction and preserves key structural elements. The synthesis and characterization of the hydrazide intermediate have been previously reported by Mateev et al. [46], supporting the reliability of the approach.

Overall, the combination of classical reactions, controlled selectivity, and functional diversity makes this synthetic route well suited for the development of novel antioxidant agents.

From an antioxidant perspective, the structural features introduced at each stage of the synthesis are significant. The pyrrole ring, hydrazide functionality, and azomethine moiety collectively contribute to electron-donating capacity and radical stabilization. Additionally, the presence of substituents derived from both the initial alkylating agent and the carbonyl partners allows fine-tuning of electronic and steric properties.

Such modularity is advantageous for structure–activity relationship (SAR) studies aimed at optimizing antioxidant performance. Furthermore, the synthetic route is adaptable and reproducible, enabling the generation of diverse analogues for biological evaluation.

### 4.2. Antioxidant Activity

For the in vitro evaluation of antioxidant potential, a combination of complementary assays—ABTS, DPPH, FRAP, and the Antioxidant Assay Kit (CS0790, Sigma-Aldrich)—was applied, selected for their reliability, reproducibility, simplicity, and rapid execution. A critical aspect in these methods is the use of an appropriate reference antioxidant; in this study, Trolox, a water-soluble analogue of vitamin E, was employed as a standard in all assays due to its well-established hydrogen atom-donating ability and widespread application in antioxidant testing systems [57]. The results are expressed as TEAC (Trolox Equivalent Antioxidant Capacity), allowing direct comparison between the tested compounds and the standard [58].

#### 4.2.1. ABTS Assay

The ABTS assay evaluates antioxidant capacity via electron transfer mechanisms, based on the reduction of the ABTS^•+^ radical cation generated by oxidation with potassium persulfate. Due to the solubility of ABTS, this method is applicable to both hydrophilic and lipophilic compounds. The decrease in absorbance at 734 nm reflects the ability of the tested compounds to act as electron donors and neutralize the radical species [59]. In the present study, compounds exhibiting strong electron-donating substituents demonstrated enhanced activity, consistent with the mechanistic basis of the assay.

#### 4.2.2. DPPH Assay

The DPPH assay is based on a hydrogen atom transfer mechanism, where antioxidants reduce the stable DPPH^•^ radical to its non-radical form (DPPH-H), accompanied by a decrease in absorbance at 517 nm [60]. This method specifically reflects radical-scavenging capacity via hydrogen donation. The results obtained correlate with the presence of functional groups capable of efficient proton transfer, particularly hydroxyl moieties.

#### 4.2.3. FRAP Assay

The FRAP assay measures the reducing power of the tested compounds through their ability to convert Fe^3+^ to Fe^2+^ in the presence of TPTZ, forming a blue-colored complex detected at 593 nm. This assay is governed by electron transfer mechanisms and provides insight into the redox potential of the compounds [61]. The observed trends align with those from the ABTS assay, confirming the importance of electron-donating substituents in enhancing antioxidant capacity.

#### 4.2.4. Antioxidant Assay Kit (CS0790) Sigma-Aldrich

This method is based on the generation of a ferryl myoglobin radical, which subsequently oxidizes ABTS to its radical cation form (ABTS^•+^), measurable at 405 nm. Antioxidants inhibit radical formation in a concentration-dependent manner, resulting in decreased absorbance. The reactions can be described as follows:

HX-Fe^III^ + H_2_O_2_ → ^•^X-[Fe^IV^=O] + H_2_O, (3)

ABTS + ^•^X-[Fe^IV^=O] → ABTS^•+^+ HX-Fe^III^, (4)

where HX-Fe^III^ represents metmyoglobin and ^•^X-[Fe^IV^=O] ferryl myoglobin. Trolox was used as a standard for comparison.

The combined results from all four assays consistently identified compounds **VV3**, **VV4**, and **VV6** as the most active antioxidants. Notably, **VV4** and **VV6** exhibited higher activity than Trolox, while **VV3** showed comparable efficacy. The antioxidant activity strongly correlates with the presence and balance of hydroxyl and methoxy substituents within the molecular structure. Hydroxyl groups function as both hydrogen and electron donors, while methoxy groups primarily contribute through electron-donating effects.

A clear structure–activity relationship is observed: **VV8**, containing a single hydroxyl group, shows low activity; **VV3**, with two hydroxyl groups, demonstrates significantly improved activity; and **VV4**, bearing three hydroxyl groups, exhibits further enhancement. The highest activity is observed for **VV6**, which contains an optimal combination of one hydroxyl and one methoxy group, suggesting a synergistic effect between hydrogen donation and electron transfer mechanisms. Conversely, **VV5**, containing two methoxy groups and one hydroxyl group, shows reduced activity, indicating that excessive substitution of hydroxyl groups with methoxy groups diminishes antioxidant potential.

Overall, these findings highlight that an optimal balance between hydroxyl and methoxy functionalities is crucial for maximizing antioxidant activity, likely due to the combined contributions of hydrogen atom transfer and electron transfer mechanisms.

### 4.3. Neuroprotection and Neurotoxicity

The neurotoxicity of the newly synthesized compounds was initially evaluated in rat brain synaptosomes by monitoring synaptosomal viability and reduced glutathione (GSH) levels. A decrease in these parameters reflects impairment of neuronal integrity and redox balance, thus serving as reliable indicators of neurotoxicity. The results demonstrate that most compounds exhibit low intrinsic toxicity, as evidenced by minimal reductions in synaptosome viability and GSH depletion.

The neuroprotective potential was further assessed using a 6-hydroxydopamine (6-OHDA)-induced toxicity model, which mimics key features of Parkinsonian neurodegeneration. The oxidative metabolism of 6-OHDA generates reactive oxygen species (ROSs) and quinone intermediates, leading to dopaminergic neuronal damage [50]. Co-treatment with the tested compounds resulted in improved synaptosome viability and attenuation of GSH depletion compared to 6-OHDA alone, indicating a protective effect against oxidative injury. These findings suggest that selected compounds can counteract ROS-mediated synaptic dysfunction, a critical factor in neurodegenerative processes.

Mitochondrial assays provided further insight into the redox-modulating properties of the compounds. Neurotoxicity was evaluated by measuring malondialdehyde (MDA) production and GSH levels following standalone application. Increased MDA formation, a marker of lipid peroxidation, together with GSH depletion, indicates oxidative damage at the mitochondrial level. The majority of compounds showed limited pro-oxidant effects, suggesting a favorable safety profile.

Neuroprotection was investigated using *tert*-butyl hydroperoxide (*t*-BuOOH)-induced oxidative stress, a widely accepted model that triggers lipid peroxidation, mitochondrial membrane disruption, and DNA damage [62]. Co-incubation with the tested compounds significantly reduced MDA levels and preserved GSH content compared to *t*-BuOOH-treated controls. These results indicate that the compounds can effectively mitigate oxidative mitochondrial damage, likely through radical scavenging and/or enhancement of endogenous antioxidant defenses.

Microsomal lipid peroxidation assays further complemented the evaluation of neurotoxicity and neuroprotection. When applied alone, the tested compounds caused minimal increases in MDA production, indicating low intrinsic toxicity. Neuroprotective activity was assessed using an iron/ascorbate (Fe^2+^/AA)-induced lipid peroxidation model. This system promotes oxidative stress through enhanced free radical generation, leading to increased MDA formation and alterations in antioxidant enzyme activities, including elevated superoxide dismutase 2 (SOD2) and reduced glutathione peroxidase (GPx) activity [63].

The tested compounds significantly decreased MDA production in the co-treatment model, demonstrating their ability to inhibit lipid peroxidation and protect membrane integrity. This effect is particularly relevant, as membrane lipid oxidation is a key event in neuronal damage and progression of neurodegenerative disorders.

The combined results from synaptosomal, mitochondrial, and microsomal models identify compounds **VV2**, **VV4**, **VV6**, and **VV9** as the most promising candidates, exhibiting both low neurotoxicity and significant neuroprotective effects. Among them, **VV4** and **VV6** show the most favorable overall profiles. Their neuroprotective activity is likely linked to their strong antioxidant properties, as demonstrated in the in vitro assays, suggesting a mechanism involving direct scavenging of reactive species and stabilization of cellular redox homeostasis. The safety profile of the newly synthesized compounds must also be taken into account, as all of them demonstrate a reduction in synaptosomal viability and GSH, as well as an increase in MDA in the experiments when they are administered alone.

Interestingly, compounds **VV2** and **VV9** exhibit notable neuroprotection despite relatively weak antioxidant activity, indicating the involvement of alternative mechanisms, such as modulation of cellular signaling pathways or interaction with specific molecular targets. Conversely, **VV3**, despite its good antioxidant capacity, shows comparatively weaker neuroprotective effects, highlighting that antioxidant activity alone is not sufficient to predict neuroprotection.

Overall, these findings emphasize the multifactorial nature of neuroprotection and the importance of integrating multiple biological models. Based on the combined antioxidant, neurotoxicity, and neuroprotection data, compounds **VV4** and **VV6** emerge as the most promising candidates for further investigation as potential neuroprotective agents. After all, the results we reached are based on in vitro models. This imposes certain limitations, as in in vivo conditions some discrepancies may be observed with the results obtained in in vitro testing. Thus, the present work is a starting point for the rational conduct of future research.

### 4.4. In Silico ADME

The pharmacokinetic behavior of compounds is an important factor for the manifestation, intensity, and duration of their pharmacological effects. The processes of absorption, distribution, metabolism, and excretion are mainly governed by the physicochemical properties of compounds. An in silico evaluation of these properties for **VV4** and **VV6** was performed using the Schrodinger/QikProp module which enables a comparison against a reference dataset of 1000 compounds, including 700 known drugs. The results show that studied derivatives possess properties similar to 95% of drugs, since their parameters fit into the reference ranges, namely MW (130.0 to 725.0), HBA (2.0 to 20.0), HBD (0.0 to 6.0), LogP (−2.0 to 6.5), and LogBB (−3.0 to 1.2). Since we are working on the creation of compounds with neuroprotective activity that could represent a perspective in the therapy of neurodegenerative diseases, the ability of the substances to cross the blood–brain barrier is of particular importance. Gastro-intestinal absorption is the other key factor in this case, as the possibility of oral administration depends on it. Due to its higher lipophylisity and lower extent of ionization at physiological pH, **VV6** is expected to show better permeability across the gastrointestinal and the blood–brain barriers. In contrast, its low predicted aquaeous solubility, falling outside the reference range for LogS (−6.5 to −0.5), and its high molecular weight which violates Lipinski’s Ro5, may lead to unfavorable pharmacokinetics. These limitations should be considered and addressed in future synthesis and development. Additionally, we need to take into account the fact that these are predicted values obtained in silico. A good prospect for future developments is the experimental determination of the pharmacokinetics of the studied compounds.

### 4.5. DFT Studies

The DFT calculations yielded data that enable the evaluation of the electronic properties and stability/reactivity of the studied compounds in relation to their potential antioxidant activity. The analysis is based primarily on the HOMO energy, the HOMO–LUMO energy gap (ΔE), ionization potential (IP), and electrophilicity index (ω), as only these parameters exhibit noticeable, albeit moderately significant, differences between compounds. The absolute HOMO and LUMO energies provide direct insight into their electron-donating ability, while HOMO–LUMO energy gap (ΔE) further indicates the overall reactivity. **VV6** exhibits a higher HOMO energy and a smaller ΔE than **VV4**, indicating enhanced electron-donating ability and greater nucleophilicity. In contrast, the larger ΔE value for **VV4** suggests that it is more stable and less chemically reactive. Additionally, **VV6** possesses lower ionization potential, meaning it requires less energy to release an electron from the ground state, which is consistent with the above observations. Although **VV6** also shows a higher electrophilicity index (ω), this does not contradict its donor character; rather, it suggests an improved capacity to stabilize electronic charge upon interaction [64].

To further support these findings, the DFT calculations were recalculated in Jaguar using the M06-2X functional with the 6-311++G(d,p) basis set, and only the HO-MO/LUMO orbitals and molecular electrostatic potential surfaces were analyzed. These recalculations confirmed the frontier-orbital distribution and charge-polarization pattern of **VV4** and **VV6**, showing that the most negative regions are concentrated around the oxygen atoms of the hydroxyl groups, which represent the most favorable sites for radical or electrophilic attack. This pattern suggests that the phenolic hydroxyl groups are the primary contributors to the antioxidant activity of **VV4** and **VV6**, since they can more readily participate in hydrogen atom transfer and subsequent radical stabilization. The frontier orbital distributions indicate that the electron density relevant to redox reactivity is delocalized over the conjugated pyrrole–hydrazone scaffold and the hydroxyl-substituted aromatic rings, which facilitates electron transfer and stabilizes the oxidized species. In this context, the phenolic hydroxyl groups are the most plausible sites for hydrogen atom transfer (HAT), while the favorable HOMO energies and small HOMO–LUMO gaps of **VV4** and **VV6** support their ability to participate in single-electron transfer (SET) processes. The molecular electrostatic potential maps further reinforce this interpretation by showing negative potential around the oxygen atoms, consistent with enhanced radical interaction and charge redistribution after oxidation. Among the studied compounds, **VV4** and **VV6** showed the most favorable electronic features, including higher HOMO energies, smaller HOMO–LUMO gaps, and more pronounced negative electrostatic potential over the hydroxylated aromatic regions, which parallel their superior antioxidant activity in the in vitro assays.

The broader distribution of negative potential over the hydroxyl-substituted aromatic regions also indicates extended charge delocalization, consistent with stabilization of the radical intermediates formed after antioxidant action. Together with the HOMO/LUMO results, the MEP maps indicate that **VV4** and **VV6** possess electronic features that favor both electron donation and stabilization of the oxidized form, helping explain their superior activity relative to the other analogues. The present DFT findings are consistent with earlier reports demonstrating that electronic descriptors, frontier orbital localization, and molecular electrostatic potential patterns can help rationalize antioxidant activity [65,66,67].

Several limitations of the present study should be considered. The antioxidant activity was evaluated only through in vitro assays, which may not fully reflect the complexity of oxidative processes under physiological conditions. Similarly, the neuroprotective effects were assessed in isolated rat brain subcellular fractions, limiting direct extrapolation to in vivo systems.

In addition, the relatively small number of synthesized derivatives restricts comprehensive structure–activity relationship analysis. The pharmacokinetic and drug-likeness properties of **VV4** and **VV6** were evaluated solely by in silico methods and require experimental validation. Furthermore, although low neurotoxicity was observed, detailed toxicological and in vivo studies are necessary to confirm the safety and therapeutic potential of the compounds.

## 5. Conclusions

In the present study, ten novel pyrrole-based hydrazide–hydrazones were synthesized and structurally characterized using spectral methods. Their antioxidant activity is evaluated by the ABTS, DPPH, FRAP assays and with the Antioxidant Assay Kit (CS0790). Among the tested compounds, **VV4** and **VV6** exhibit the highest antioxidant activity, exceeding that of the reference standard, Trolox. The neurotoxic and neuroprotective potential of the synthesized compounds is further assessed in rat brain synaptosomes, mitochondria, and microsomes. Compounds **VV2**, **VV4**, **VV6**, and **VV9** demonstrate the most pronounced neuroprotective effects while maintaining relatively low neurotoxicity. As the most promising candidates, **VV4** and **VV6** are further subjected to in silico characterization of their pharmacokinetic behavior and drug-likeness, as well as to DFT analysis for the evaluation of their electronic properties and chemical reactivity. These analyses identify **VV6** as the most promising derivative, owing to its more favorable predicted gastrointestinal and blood–brain barrier permeability, along with its enhanced electron-donating ability and overall reactivity. The study also outlines future directions aimed at optimizing molecular weight and improving solubility.

## Figures and Tables

**Table 1:** IC_50_ values of the tested compounds from the ABTS test.

Compound	IC_50_ (μM ± SD)
**VV1**	>1000
**VV2**	455.43 ± 0.19
**VV3**	16.87 ± 0.07
**VV4**	12.23 ± 0.17
**VV5**	347.09 ± 0.20
**VV6**	8.93 ± 0.22
**VV7**	331.76 ± 0.23
**VV8**	>1000
**VV9**	>1000
**VV10**	>1000
**Trolox**	17.04 ± 0.26

**Table 2:** IC_50_ values of the tested compounds from the DPPH test.

Compound	IC_50_ (μM ± SD)
**VV1**	>1000
**VV2**	>1000
**VV3**	38.03 ± 0.09
**VV4**	27.46 ± 0.12
**VV5**	665.24 ± 0.20
**VV6**	24.32 ± 0.08
**VV7**	>1000
**VV8**	>1000
**VV9**	>1000
**VV10**	>1000
**Trolox**	31.92 ± 0.12

**Table 3:** TEAC values of the tested compounds from the conducted FRAP test.

Compound	TEAC Ratio ± SD
**VV1**	0.06 ± 0.01
**VV2**	0.08 ± 0.02
**VV3**	1.34 ± 0.03
**VV4**	1.73 ± 0.06
**VV5**	0.13 ± 0.01
**VV6**	2.93 ± 0.04
**VV7**	0.19 ± 0.02
**VV8**	0.06 ± 0.01
**VV9**	0.05 ± 0.01
**VV10**	<0.01

**Table 4:** TEAC values of the tested compounds from the Antioxidant Assay Kit (CS0790) Sigma-Aldrich test.

Compound	TEAC Ratio ± SD
**VV1**	<0.01
**VV2**	0.05 ± 0.01
**VV3**	1.19 ± 0.03
**VV4**	1.43 ± 0.06
**VV5**	0.11 ± 0.01
**VV6**	2.56 ± 0.03
**VV7**	0.14 ± 0.02
**VV8**	<0.01
**VV9**	<0.01
**VV10**	<0.01

**Table 5:** Predicted values of the most important physicochemical parameters of **VV4** and **VV6**. Reference ranges for parameters, calculated by Schrodinger/QikProp are given below the table.

Cmpd	MW	HBA	HBD	LogP	LogS	LogBB	HOA	pKa	LogD	f_A_	Ro5
**VV4**	558.42	4	7	4.02	−6.773	−0.882	69%	7.54	3.78	0.42	Yes 1 viol.
**VV6**	556.45	2	6	4.811	−7.677	−0.678	100%	9.18	4.8	0.02	Yes 1 viol.

Molecular weight (MW) (Range: 130.0 to 725.0); hydrogen bond acceptors (HBA) (range from 2.0 to 20.0); hydrogen bond donors (HBD) (range from 0.0 to 6.0); octanol/water partition coefficient (LogP) (range from −2.0 to 6.5); predicted aqueous solubility (LogS) (range from −6.5 to −0.5); predicted brain/blood partition coefficient (LogBB) (range from −3.0 to 1.2).

**Table 6:** Main electronic properties of compounds **VV4** and **VV6** from the DFT study.

Comd	E Total (Hartree)	E HOMO (eV)	E LUMO (eV)	ΔE (eV)	IP (eV)	A (eV)	μ (eV)	η (eV)	χ (eV)	S (eV)	ω (eV)	ΔN_max_
**VV4**	−113,836.73	−6.32	−2.11	4.21	6.32	2.11	−4.22	2.11	4.22	0.24	4.22	1.00
**VV6**	−112,861.83	−6.21	−2.18	4.03	6.21	2.18	−4.20	2.02	4.20	0.25	4.37	1.04

The highest occupied molecular orbital (HOMO), the lowest unoccupied molecular orbital (LUMO), energy gap between LUMO and HOMO energies (ΔE), ionization potential (IP), electron affinity (A), chemical potential (µ), hardness (η), Mulliken electronegativity (χ), softness (S), electrophilicity index (ω), maximum charge transfer (∆Nmax).

## Data Availability

The original contributions presented in this study are presented in the article. Further inquiries can be directed to the corresponding author.

## Abbreviations

The following abbreviations are used in this manuscript:

6-OHDA	6-hydroxydopamine
AA	ascorbic acid
ABTS	2,2′-azino-bis(3-ethylbenzothiazoline-6-sulfonic acid)
ABTS·^+^	ABTS radical cation
AD	Alzheimer’s disease
ADME	absorption, distribution, metabolism and excretion
ALS	amyotrophic lateral sclerosis
CNS	central nervous system
DFT	density functional theory
DPPH	2,2-Diphenyl-1-picrylhydrazyl
FRAP	ferric reducing antioxidant power
GSH	reduced glutathione
HD	Huntington’s disease
HOMO	highest occupied molecular orbital
IR	infrared spectroscopy
LUMO	lowest unoccupied molecular orbital
MDA	malondialdehyde
NMR	nuclear magnetic resonance
PD	Parkinson’s disease
t-BuOOH	tert-Butyl hydroperoxide
TLC	thin-layer chromatography
